# Computational Prediction and Experimental Assessment of Secreted/Surface Proteins from *Mycobacterium tuberculosis* H37Rv

**DOI:** 10.1371/journal.pcbi.1000824

**Published:** 2010-06-24

**Authors:** Carolina Vizcaíno, Daniel Restrepo-Montoya, Diana Rodríguez, Luis F. Niño, Marisol Ocampo, Magnolia Vanegas, María T. Reguero, Nora L. Martínez, Manuel E. Patarroyo, Manuel A. Patarroyo

**Affiliations:** 1 Fundación Instituto de Inmunología de Colombia – FIDIC, Bogotá, Colombia; 2 Microbiology postgraduate program, Universidad Nacional de Colombia, Bogotá, Colombia; 3 School of Medicine and Health Sciences, Universidad del Rosario, Bogotá, Colombia; 4 Intelligent Systems Research Laboratory – LISI, Universidad Nacional de Colombia, Bogotá, Colombia; 5 Research Group on Combinatorial Algorithms – ALGOS-UN, Universidad Nacional de Colombia, Bogotá, Colombia; 6 Instituto de Biotecnología – IBUN, Universidad Nacional de Colombia, Bogotá, Colombia; 7 School of Medicine, Universidad Nacional de Colombia, Bogotá, Colombia; University of California San Diego, United States of America

## Abstract

The mycobacterial cell envelope has been implicated in the pathogenicity of tuberculosis and therefore has been a prime target for the identification and characterization of surface proteins with potential application in drug and vaccine development. In this study, the genome of *Mycobacterium tuberculosis* H37Rv was screened using Machine Learning tools that included feature-based predictors, general localizers and transmembrane topology predictors to identify proteins that are potentially secreted to the surface of *M. tuberculosis*, or to the extracellular milieu through different secretory pathways. The subcellular localization of a set of 8 hypothetically secreted/surface candidate proteins was experimentally assessed by cellular fractionation and immunoelectron microscopy (IEM) to determine the reliability of the computational methodology proposed here, using 4 secreted/surface proteins with experimental confirmation as positive controls and 2 cytoplasmic proteins as negative controls. Subcellular fractionation and IEM studies provided evidence that the candidate proteins Rv0403c, Rv3630, Rv1022, Rv0835, Rv0361 and Rv0178 are secreted either to the mycobacterial surface or to the extracellular milieu. Surface localization was also confirmed for the positive controls, whereas negative controls were located on the cytoplasm. Based on statistical learning methods, we obtained computational subcellular localization predictions that were experimentally assessed and allowed us to construct a computational protocol with experimental support that allowed us to identify a new set of secreted/surface proteins as potential vaccine candidates.

## Author Summary

Since the publication of the *Mycobacterium tuberculosis* genome in 1998, great expectations have emerged regarding speeding up the process of developing vaccines against tuberculosis. Our group has been focused on identifying molecules localized on the mycobacterial surface that could act as ligands facilitating this pathogen's entry into host cells. Immune responses exerted against these proteins might block receptor-ligand interactions, thus hampering mycobacterial invasion. Since protein fragments involved in these interactions might serve as vaccine candidates and, taking into account that a relatively small number of mycobacterial surface proteins have been experimentally identified to date due to the inherent difficulty of proteomics studies for characterizing surface proteins, in this study, we used Machine Learning-based tools available on the World Wide Web to obtain accurate predictions of surface and secreted proteins from this pathogen and found experimental support of such predictions for a group of candidate proteins selected based on novel criteria.

## Introduction

According to the Statistics reported by the World Health Organization, *Mycobacterium tuberculosis* causes 9.27 million new cases of tuberculosis (TB) each year and approximately 1.7 million deaths among infected people worldwide [1]. Host–pathogen interactions leading to mycobacterial infection are mediated by a variety of cell receptor ligands, signal transduction proteins and enzymes, among others [2], [3]. A large number of these molecules are exposed on the surface of the tuberculous bacillus where they are in direct contact with the host's cells and likely to play key roles in the initial stages of the bacillus invasion, virulence, pathogenesis and survival inside host cells [4]. This has led to focusing most vaccine and drug development efforts on the identification of mycobacterial cell surface and secreted proteins, a goal that has been enormously facilitated by the publication of the complete genomic sequence of *M. tuberculosis* (H37Rv strain). However, despite the large amount of data available, the structure, function and localization of a large number of hypothetical or putative proteins have not been yet defined [5], [6], mainly due to methodological difficulties related to proteomic and transcriptomic analyses [7].

Secretion of mycobacterial proteins to the membrane and the extracellular milieu is tightly regulated through different secretory routes or pathways [8], [9]. In bacteria one of the best characterized secretory systems is the Sec-dependent pathway (or classical pathway), which transports unfolded protein precursors to the cell membrane [10]–[12]. There are other protein secretion mechanisms alternative to the classical secretory pathway such as the twin-arginine translocation (Tat) and lipoprotein secretion pathways. Tat has been extensively studied in *Escherichia coli* (Gram-negative bacteria) and *Bacillus subtilis* (Gram-positive bacteria) where it is known to mediate secretion of folded proteins containing a conserved consensus N-terminal sequence. In contrast, proteins that are targeted to the lipoprotein secretion pathway contain a consensus sequence known as the “lipobox” motif at the signal sequence's C-terminal portion [13]–[15], which drives prolipoprotein maturation and lipoprotein anchoring to the cell surface [16]. Other proteins are secreted through different mechanisms despite not containing a distinguishable secretion signal sequence and are commonly referred to as non-classically secreted proteins [17]–[19].

A fundamental goal of cell biology is to define the functions of proteins in the context of the compartments inside which they are organized within the cellular environment [20]. To facilitate making inferences regarding protein function, annotating genomes and designing proteomics experiments [21], [22], proteins reported in the Swiss-Prot protein database [23] have been categorized according to their localization into cytoplasmic, membrane-anchored, cell wall, and extracellular proteins (lipid anchored, secreted and bacteriocine-like proteins) [24].

The identification of both the secretory mechanisms and the subcellular localization of proteins are supported by two computational strategies commonly referred to as featured-based and general localization approaches, respectively; a distinction made based on the different computational protocols available for protein selection [25]. The first approach is based on sequence pattern or motif recognition, while the second one consists in extracting general amino acid profiles. These two approaches can be combined to postulate better subcellular localization/secretion predictions with the aim of identifying secreted/surface proteins.

Bearing in mind this idea and that only a reduced number of mycobacterial surface proteins are currently annotated, this study was divided into two phases: (a) a computational and (b) an experimental phase which was meant to validate the first phase.

In the computational phase, the genome of *M. tuberculosis* H37Rv [5] was screened with Machine Learning tools (feature-based tools, general localizers and transmembrane topology predictors) to identify proteins that are likely secreted to the mycobacterial surface or the extracellular milieu. The first approach involved the use of the featured-based tools SignalP 3.0 [26], TatP 1.0 [27], LipoP 1.0 [28] and SecretomeP 2.0 [29], while the general localization predictors PA-SUB version 2.5 included in Proteome Analyst 3.0 [30], Gpos-PLoc [22] and PSORTb version 2.0.4 [21] were used for the second approach.

The mycobacterial cell envelope has a complex architectural rearrangement with three well differentiated structures of unique composition. The inner layer of plasma membrane is composed mainly of cardiolipin, phosphatidylglycerol, phosphatidylethanolamine, and phosphatidyl mannosides, which are precursors of lipoarabinomanan (LAM). The middle layer is the cell wall that is mainly composed of free lipids, proteins and peptidoglycan, which is covalently bonded to one molecule of arabinogalactan and esterified with mycolic acids. Finally, the capsule or outer layer is composed of polysaccharides such as mannose and arabinomannan, proteins and lipids [31].

Some of the most distinctive morphological characteristics of mycobacteria are precisely due to the composition and structure of the mycobacterial cell envelope, including resistance to decoloration by acid alcohol in Gram staining, its characteristic “strings and tangles” appearance of growth, resistance to drying and to treatments with alkali and physical and chemical agents [32]. Particularly, the resistance of mycobacteria to the most commonly used antimicrobial agents is attributable to the low permeability of the mycobacterial cell envelope, a characteristic that is conferred essentially by the cell wall layer of mycolic acids [33]. Given its complexity, the Machine Learning tools used in this study were first validated regarding their predictive capacity to classify mycobacterial proteins [34], and the transmembrane topology of the potentially secreted/surface proteins was determined using TMHMM 2.0 [35] and Phobius [36]. The aim of this study was to determine, based on experimental methodologies, that secreted/surface mycobacterial proteins could be identified using computational approaches.

For such purpose, a set of hypothetically secreted/surface mycobacterial candidate proteins were chosen based on the following criteria: (a) prediction of secretion by only one of the four feature-based tools with a probability score of ≥0.5 [25]–[29], (b) prediction of cytoplasmic or extracellular localization by at least 2 of the 3 general localizers, (c) prediction of more than one transmembrane helix, preferably not located in the N-terminal region, (d) preferably having no experimental confirmation of secretion/surface localization (a special case occurred with proteins predicted proteins by SecretomeP 2.0 and LipoP 1.0), and (e) not being included within the training sets of any of the Machine-Learning Tools used in this study.

Four proteins were selected from the set of secreted/surface proteins experimentally identified by Gu *et al.* [37] and Sinha *et al.* [38] as positive controls, while two proteins were selected from the set of cytoplasmic proteins reported in the TB Structural Genomics Consortium (TBsgc) protein database as negative controls [6]. Once these the final protein sets had been defined as shown in Table 1, linear B cell epitopes were predicted using classical methods that assign values to each amino acid according to their physicochemical properties and therefore allowed selecting peptides based on inferences made regarding their probable antigenic activity [39], [40]. Such peptides were synthesized as polymers and were inoculated into rabbits to obtain specific antisera that were used in the experimental phase to assess protein localization by immunoblotting analysis of mycobacterial subcellular fractions and IEM studies so as to evaluate the accuracy of the computational predictions.

**Table 1:** Number of proteins in each of the protein sets explored in this study.

	Localization	Source	*N*	*n*
Candidate proteins	hypothetically secreted/surface	*M. tuberculosis* H37Rv genome	3,924	8
Positive control proteins	secreted/surface	Gu *et al.* and Sinha *et al.*	100	4
Negative control proteins	cytoplasmic	TBsgc	9	2

*N*: total number of proteins in the raw data sets explored in the computational phase of the study. Feature-based, general localization and transmembrane topology predictions obtained for each protein are shown in Tables S1, S2, S3 and S4 (*M. tuberculosis* H37Rv genome ORFs), Tables S5 (proteins reported by Gu *et al.* and Sinha *et al.*) and S6 (cytoplasmic proteins reported in TBsgc: TB Structural Genomics Consortium).

*n*: number of proteins from each protein set source selected based the criteria established in this study whose subcellular localization was assessed in the experimental phase.

## Results

### Computational phase

The computational screening of the 3,924 open reading frames (ORFs) reported in the *M. tuberculosis* H37Rv genome with the feature-based tools reported a total of 825 proteins that are likely secreted to the extracellular milieu or to the mycobacterial surface. These 825 proteins were independently predicted by a single feature-based tool (each representing a different secretory mechanism) with a probability score of ≥0.5 as follows: 162 proteins were exclusively predicted by SignalP 3.0, 106 only by TatP 1.0, 1 only by LipoP 1.0 and 556 only by SecretomeP 2.0. These proteins were then screened with the general localizers and transmembrane topology predictors and 2 candidate proteins were chosen per secretory mechanism with the aim of assessing their localization in the experimental phase. Candidate proteins were selected based on the following criteria: they had to contain at least one predicted transmembrane helix located preferably outside the N-terminal region. Secretion of the candidate proteins to the cytoplasmic membrane or the extracellular milieu had to be predicted by at least two of the general localizers. Preferably, candidate proteins should not have been located in the surface of *M. tuberculosis* by experimental methodologies yet and should not be part of the training datasets of the Machine-Learning Tools used in this study.

According to such criteria, the following candidate proteins were chosen from the sets of proteins predicted by each of the feature-based tools: Rv0403c and Rv1733c from the set of proteins predicted by SignalP 3.0, Rv3069 and Rv3630 from the set predicted by TatP 1.0, Rv1022 and Rv0835 (the latter also predicted by SignalP 3.0 in our computational analysis and identified by Malen *et al.* [41] as an exported protein using proteomics) from the set of proteins predicted by LipoP 1.0, and finally Rv0361 and Rv0178 from the set of proteins predicted by SecretomeP 2.0 (Table 2).

**Table 2:** Results of the computational and experimental phases for the candidate proteins, positive and negative controls.

	Computational phase											Experimental phase		
	Proteins	Cleavage site		PA-SUB v.2.5			Gpos-PLoc	PSORTb v. 2.0.4	TMHMM 2.0 (PredHel)	Phobius		TPS	SF	IEM
		Score	Position	C	E	PM				TM	SP			
**SignalP 3.0**	**Rv0200 (+)**	0.885	between positions 23 and 24	−	−	+	plasma membrane	cytoplasmic membrane	3	3	−	+	+	+
	**Rv1733c**	0.974	between positions 62 and 63	−	−	+	plasma membrane	unknown	2	2	−	−	−	NA
	**Rv0403c**	0.974	between positions 33 and 34	−	−	+	plasma membrane	cytoplasmic membrane	2	0	+	+	+	+
**TatP 1.0**	**Rv1280c (+)**	0.819	between positions 41 and 42: ATA-GA	−	+	−	cytoplasm	unknown	1	0	+	+	−	+
	**Rv3069**	0.853	between positions 30 and 31: ALA-IP	−	−	+	plasma membrane	cytoplasmic membrane	4	3	+	−	−	NA
	**Rv3630**	0.813	between positions 39 and 40: GTA-AA	−	−	+	plasma membrane	cytoplasmic membrane	11	12	−	+	−	+
**LipoP 1.0**	**Rv0418 (+)**	0.954	between positions 24–25: FLTTG-CIRWS Pos+2 = I	−	+	−	cytoplasm	extracellular	1	0	+	+	−	+
	**Rv1022**	0.941	between positions 23–24: LLASS-CTWQL Pos+2 = T	+	−	−	cytoplasm	unknown	1	0	+	−	+	+
	**Rv0835**	11.3	between positions 22–23: IATTA-CSFQA Pos+2 = S	−	−	+	cytoplasm	cytoplasmic	0	0	+	+	+	+
**SecretomeP 2.0**	**Rv0556 (+)**	0.829	NA	−	−	+	plasma membrane	cytoplasmic membrane	2	2	−	+	−	+
	**Rv0361**	0.924	NA	−	−	+	cytoplasm	unknown	1	1	−	+	+	+
	**Rv0178**	0.790	NA	−	−	+	extracellular	unknown	1	1	−	+	+	+
**Negative controls**	**Rv0126 (−)**	NA	NA	+	−	−	cytoplasm	cytoplasmic	0	0	−	+	−	+
	**Rv1326c (−)**	NA	NA	+	−	−	cytoplasm	cytoplasmic	0	0	−	−	−	NA

PredHel: number of transmembrane helices predicted by TMHMM 2.0; (+): positive control proteins, (−): negative control proteins; PA-SUB v.2.5 predictions are classified as: NA: Not applicable. C: cytoplasmic; E: extracellular; PM: plasma membrane. In Phobius predictions: TM indicates the number of predicted transmembrane segments, SP: indicates if there is a predicted signal peptide. TPS: Protein recognition by the specific immune sera in *M. tuberculosis* H37Rv total protein sonicate; SF: Protein recognition by the specific immune sera in *M. tuberculosis* H37Rv membrane and culture filtrate (for positive controls and candidate proteins) or cytoplasm (for negative controls) by subcellular fractionation. IEM: Protein recognition on the surface of intact *M. tuberculosis* H37Rv bacillus by immune electron microscopy. None of these proteins was part of the training data sets of any of the Machine Learning tools.

It should be taken into account that in case of the proteins predicted by SecretomeP 2.0, Rv0361 was identified by Xiong *et al.* as an integral membrane protein using one-dimensional (1D) gel electrophoresis and liquid chromatography electrospray ionization tandem mass spectrometry (MS) [42], and Rv0178 was identified by Gu *et al.* in membrane fraction by 1D sodium dodecyl sulfate polyacrylamide gel electrophoresis (SDS-PAGE) coupled with microcapillary liquid chromatography LC-MS/MS [37]. These two proteins were chosen as candidates to be assessed in the experimental phase despite having experimental confirmation of surface localization, because they could be used to evaluate the accuracy of SecretomeP 2.0 predictions regarding the non-classical secretion problem. As a result, three of the secreted/surface candidate proteins had been experimentally confirmed by other authors (without the use of bioinformatics tools) while the localization of the other 3 proteins had not been experimentally determined previously.

The same screening over the 100 proteins reported Gu *et al.* [37] and Sinha *et al.* [38] identified 84 as possible secreted/surface proteins. Six of these proteins were only predicted by SignalP 3.0, 1 protein only by TatP 1.0, none by LipoP 1.0, and 48 only by SecretomeP 2.0. On the basis of the screening done with the general localizers and the topology predictors, Rv0200, Rv1280c and Rv0556 were chosen to be assessed in the experimental phase as positive controls of SignalP 3.0, TatP 1.0 and SecretomeP 2.0 predictions, respectively (Table 2). Since no proteins were exclusively predicted by LipoP 1.0, Rv0418 (predicted also by Signal 3.0 and SecretomeP 2.0) was selected as the positive control for LipoP 1.0. On the other hand, Rv0126 [43]–[45] and Rv1326c [46], [47] were selected as negative controls based on previously described reports. The same computational screening was applied to positive and negative controls to compare predictions on these proteins with predictions on the candidate proteins (Table 2).

The results of the computational screening with the feature-based tools, the general localizers and the transmembrane topology predictors on the complete genome of *M. tuberculosis* H37Rv, from which the candidate proteins were selected, are shown discriminated by feature-based tool in Tables S1, S2, S3 and S4, while the results for the 100 proteins experimentally identified by Gu *et al.* [37] and Sinha *et al.* [38] from which positive controls were selected are shown in Table S5 and the screening on the cytoplasmic proteins reported in TBsgc from which negative controls were selected are shown in Table S6.

The transition from the computational phase to the experimental phase consisted on predicting linear B cell epitopes on the candidate, positive and negative proteins using ANalyse THE PROTeins (AntheProt) [48] and BepiPred 1.0 [39]. These epitopes were synthesized and inoculated into rabbits to obtain antisera specific against each protein. Rabbit hyperimmune sera were used in immunoblotting assays to assess the presence of each protein in *M. tuberculosis* sonicate and its localization by IEM. Peptide sequences and each protein's molecular weight are shown in Table 3. It is important to mention that proteins that are labeled with an asterisk in this table correspond to proteins for which sera were already available in the FIDIC's sera collection and therefore were not subjected to the prediction of linear B cell epitopes.

**Table 3:** Sequences of the linear B cell epitopes synthesized for candidate proteins, positive and negative controls, molecular weights expected and observed in the immunoblotting assays.

	Computational phase			Experimental phase
	Peptide sequence	Monomer length (aa)^a^	E (kDa)	O (kDa)
* **Rv0200 (+)**	LVLLVVEGVAINFWLLRRD	19	24.032	26.2
	QAARALRVTLTKRGSGWLV	19		
* **Rv1733c**	AAAGTAVQDSRSHVY	15	22.461	ND
	TATSAPPRTKITVPARWVVNY	21		
* **Rv0403c**	AAVTVSRLHSVFGSHQHAPD	20	15.262	15.74
	VIREERIVNAYHAHTSTLVKSA	22		
**Rv1280c (+)**	ALRASFQGKSRPWTQTRYWA	20	63.484	65.6
	DGYQDNSGVVAYNPEQAKRE	20		
**Rv3069**	AALSALAIPDPARWPWPTFT	20	14.303	ND
	MPNHDYRELAAVFAGGALGA	20		
**Rv3630**	ADGRRTHPLRVSGMVGLGSL	20	43.491	45.2
	LTRAPLLVPLTAMQGNLIAH	20		
**Rv0418 (+)**	LKMAGKTAQDTSFDGRSDYD	20	52.049	51.92
	VAAPADDSPGCSPSDYDRLP	20		
* **Rv1022**	ASSTTWQLSLFITDGVPPPP	20	25.835	ND
	DGIANVDNIDDAALSAAGYL	20		
* **Rv0835**	VDSLIVSIEDVRRIANYEEL	20	22.905	22.81
	LDRPDASTVRIGAAGWSHVY	20		
* **Rv0556 (+)**	TRGRIVLRWLRIAVLIVTGL	20	18.759	17.96
	TPDRITYRPQLGVLYPSELS	20		
* **Rv0361**	DAETETVVITTSDNDAAVTQ	20	29.986	29.0
	RSLDLQFRDDQWKITQSSSN	20		
* **Rv0178**	STDTASAATEGHRGEIDAAG	20	25.915	24.4
	AVESLSGRDAVAIVYTNTTT	20		
**Rv1326c (−)**	MSRSEKLTGEHLAPEPAEMA	20	81.739	ND
	RFDGTPLYEHSDPKRGEQLD	20		
**Rv0126 (−)**	TSERYTDARIIFVDTEESNW	20	68.601	68.9
	VTDEERDYMYAEYAKDPRMK	20		

E: expected molecular weight based on sequence (in kDa). O: observed molecular weight (kDa) in immunoblotting assays.

*Proteins for which sera were already available and therefore did not undergo B cell prediction.

a Monomer lengths do not include the cysteine and glycine (CG) residues added at the peptide's ends to enable polymerization.

ND: no data are available for proteins that were not recognized in *M. tuberculosis* H37Rv TPS by the immune sera.

### Experimental phase

The localization of the candidate, positive and negative control proteins was assessed using sera raised against linear B cell epitope polymers of each protein.

**Figure 1:**
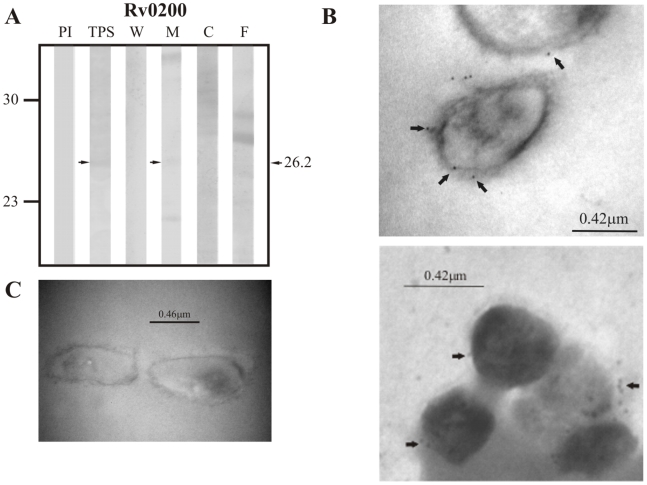
Experimental assessment of the subcellular localization of Rv0200, which was selected as positive control protein for surface/secreted predictions of SignalP 3.0. (A) Immunoblotting assessment of the presence of Rv0200 in TPS: total protein sonicate, W: cell wall, M: membrane, C: cytosol and F: culture filtrate of *M. tuberculosis* H37Rv, using specific antisera raised in rabbits. PI: assessment of the pre-immune serum showing no recognition of any mycobacterial protein. Molecular weight marker is shown on the left (P7709S ColorPlus Prestained Protein Marker, New England Biolabs) and the molecular weight observed for Rv0200 is shown to the right. (B) IEM assessment of the presence of Rv0200 on the surface of intact *M. tuberculosis* H37Rv bacilli (magnification: 40,000×). Proteins detected by anti-rabbit antibody conjugated to 10-nm colloidal gold particles are indicated by the black arrows. (C) Pre-immune serum showed no recognition of any mycobacterial proteins. The results showed detection of Rv0200 in TPS, membrane and surface of *M. tuberculosis* H37Rv.

**Figure 2 pcbi-1000824-g002:**
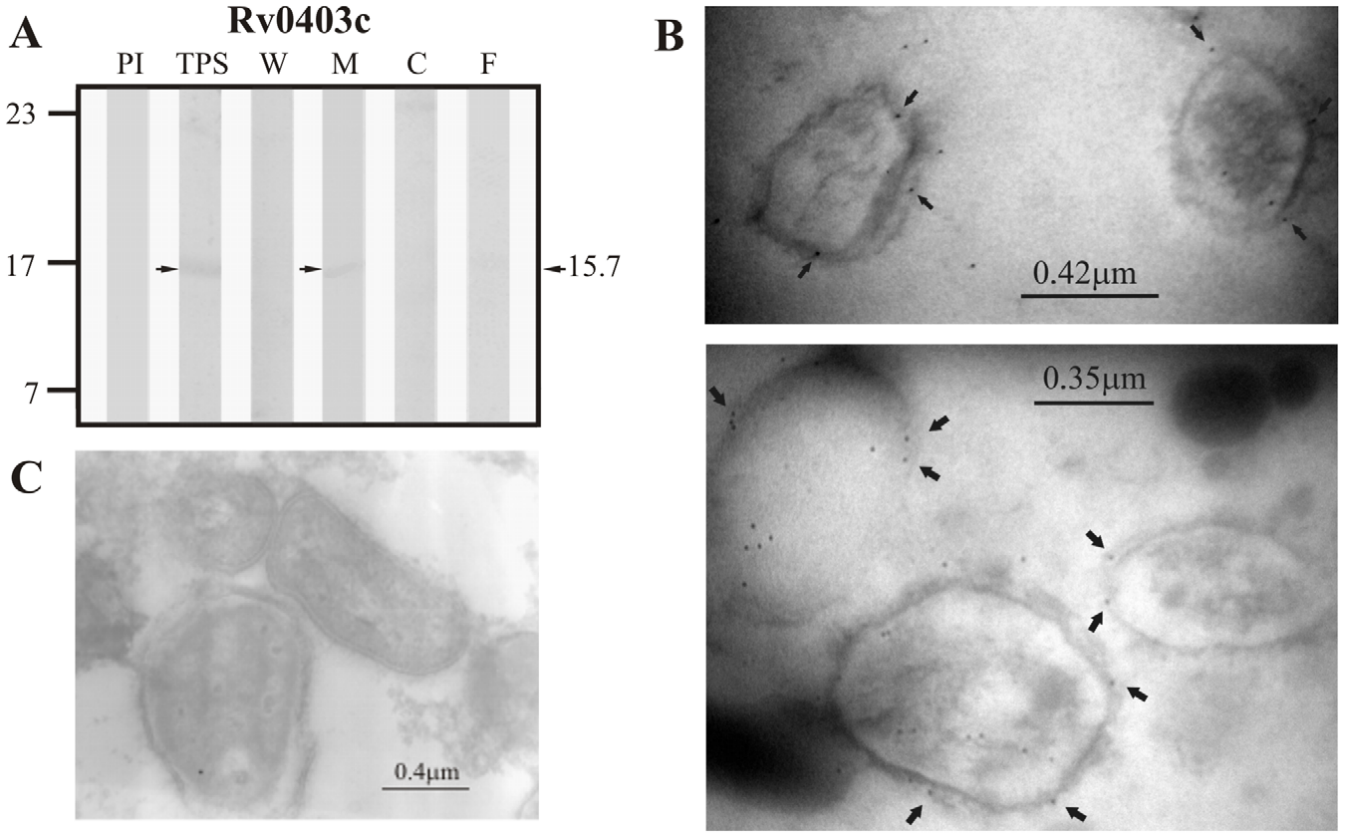
Experimental assessment of the subcellular localization of the candidate protein Rv0403c predicted as a surface/secreted by SignalP 3.0. (A) Immunoblotting assessment of the presence of Rv0403c in TPS: total protein sonicate, W: cell wall, M: membrane, C: cytosol and F: culture filtrate of *M. tuberculosis* H37Rv, using specific antisera raised in rabbits. PI: assessment of the pre-immune serum showing no recognition of any mycobacterial protein. Molecular weight marker is shown on the left (P7709S ColorPlus Prestained Protein Marker, New England Biolabs) and the molecular weight observed for Rv0403c is shown to the right. (B) IEM assessment of the presence of Rv0403c on the surface of intact *M. tuberculosis* H37Rv bacilli (magnification: 40,000×). Proteins detected by anti-rabbit antibody conjugated to 10-nm colloidal gold particles are indicated by the black arrows. (C) Pre-immune serum showed no recognition of any mycobacterial proteins. The results showed detection of Rv0403c in TPS, membrane and surface of *M. tuberculosis* H37Rv.

Regarding proteins that were predicted to be secreted via the classical secretion pathway by SignalP 3.0, sera raised against the positive control Rv0200 detected a band of ∼26.2 kDa in total protein sonicate (TPS) and in the membrane fraction (Figure 1A), which is consistent with the expected molecular weight of this protein (24.0 kDa). Rv0403c, one of the candidate proteins for this pathway was also detected in TPS and the membrane fraction by its specific hyperimmune sera at ∼15.74 kDa, which is close to this protein's theoretical molecular weight of 15.3 kDa (Figure 2A). No proteins were recognized by rabbit antisera raised against Rv1733c, the second candidate protein for this pathway (expected molecular weight of 22.5 kDa).

**Figure 3 pcbi-1000824-g003:**
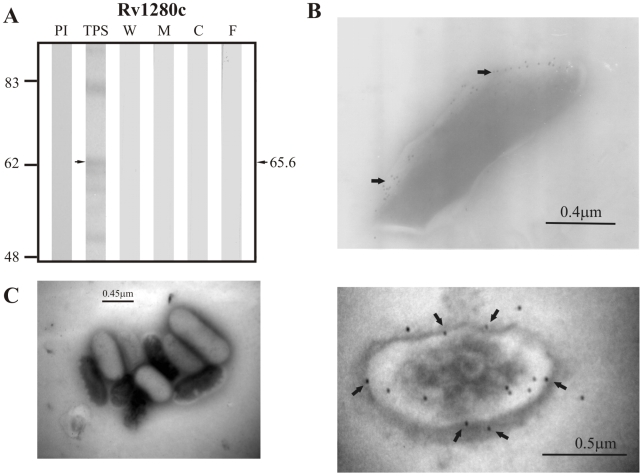
Experimental assessment of the subcellular localization of protein Rv1280c, which was selected as positive control protein for surface/secreted predictions of TatP 1.0. (A) Immunoblotting assessment of the presence of Rv1280c in TPS: total protein sonicate, W: cell wall, M: membrane, C: cytosol and F: culture filtrate of *M. tuberculosis* H37Rv, using specific antisera raised in rabbits. PI: assessment of the pre-immune serum showing no recognition of any mycobacterial protein. Molecular weight marker is shown on the left (P7708S ColorPlus Prestained Protein Marker, New England Biolabs) and the molecular weight observed for Rv1280c is shown to the right. (B) IEM assessment of the presence of Rv1280c on the surface of intact *M. tuberculosis* H37Rv bacilli (magnification: 40,000×). Proteins detected by anti-rabbit antibody conjugated to 10-nm colloidal gold particles are indicated by the black arrows. (C) Pre-immune serum showed no recognition of any mycobacterial proteins. The results showed detection of Rv1280c in TPS and surface of *M. tuberculosis* H37Rv.

**Figure 4 pcbi-1000824-g004:**
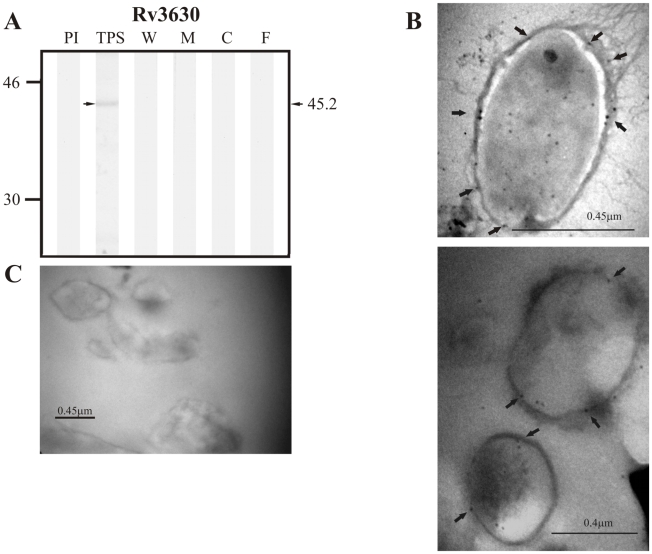
Experimental assessment of the subcellular localization of the candidate protein Rv3630 predicted as a surface/secreted by TatP 1.0. (A) Immunoblotting assessment of the presence of Rv3630 in TPS: total protein sonicate, W: cell wall, M: membrane, C: cytosol and F: culture filtrate of *M. tuberculosis* H37Rv, using specific antisera raised in rabbits. PI: assessment of the pre-immune serum showing no recognition of any mycobacterial protein. Molecular weight marker is shown on the left (P7708S ColorPlus Prestained Protein Marker, New England Biolabs) and the molecular weight observed for Rv3630 is shown to the right. (B) IEM assessment of the presence of Rv3630 on the surface of intact *M. tuberculosis* H37Rv bacilli (magnification: 40,000×). Proteins detected by anti-rabbit antibody conjugated to 10-nm colloidal gold particles are indicated by the black arrows. (C) Pre-immune serum showed no recognition of any mycobacterial proteins. The results showed detection of Rv3630 in TPS and surface of *M. tuberculosis* H37Rv.

With respect to proteins predicted to be secreted via the twin-arginine translocation pathway according to TatP 1.0, the positive control protein Rv1280c was detected in TPS as a band of ∼65.6 kDa close to the expected molecular weight of 63.5 kDa but was not detected in any of the subcellular fractions or in culture filtrate (Figure 3A). Similarly, the candidate protein Rv3630 was detected in TPS at ∼45.2 kDa (theoretical molecular weight: 43.5 kDa), but was not observed in any of the subcellular fractions nor in culture filtrate (Figure 4A). Antisera raised against Rv3069, the other candidate protein for this pathway, did not recognize this protein in TPS nor in any of the subcellular fractions (expected molecular weight of 14.3 kDa).

**Figure 5 pcbi-1000824-g005:**
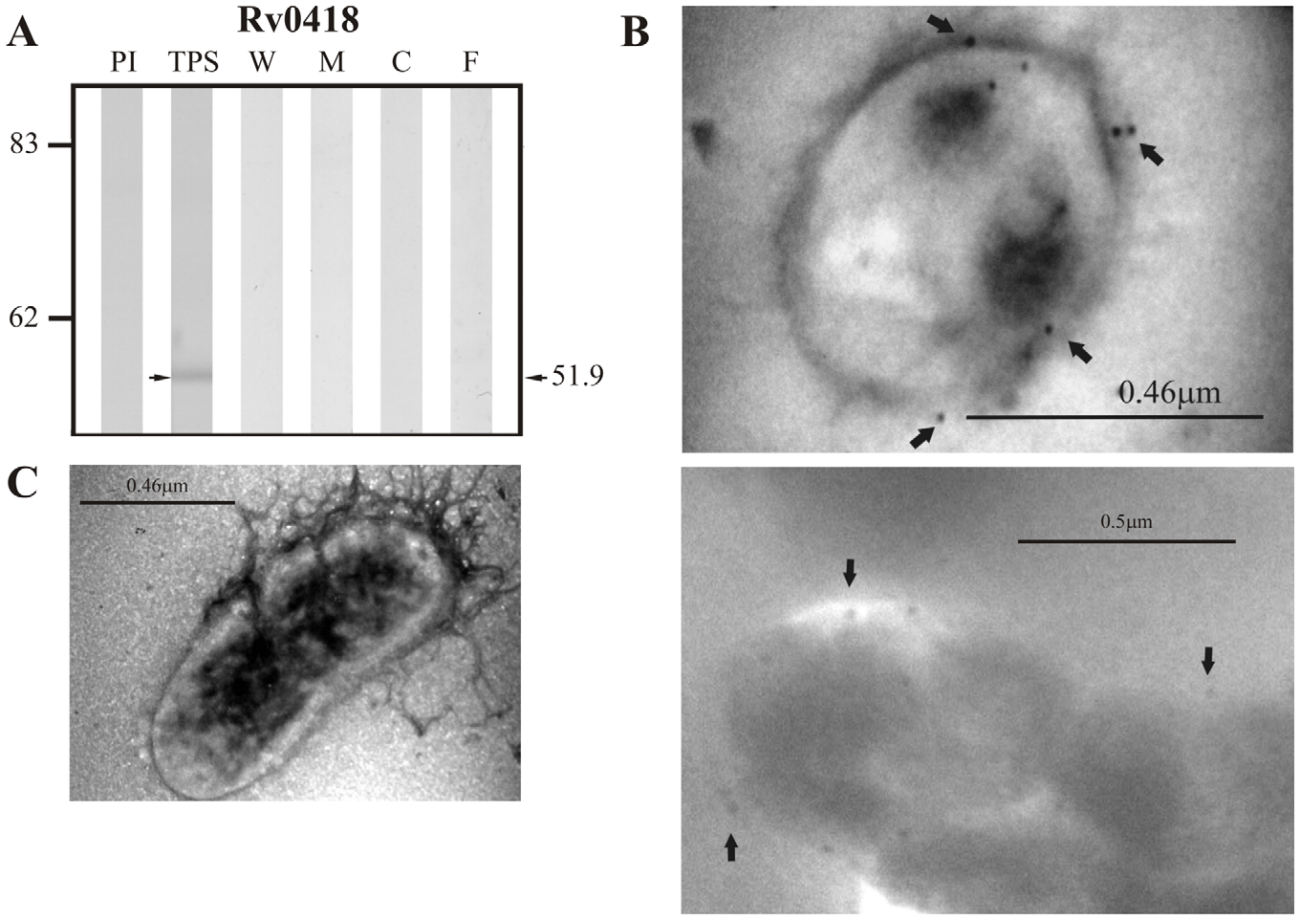
Experimental assessment of the subcellular localization of Rv0418, which was selected as positive control protein for surface/secreted predictions of LipoP 1.0. (A) Immunoblotting assessment of the presence of Rv0418 in TPS: total protein sonicate, W: cell wall, M: membrane, C: cytosol and F: culture filtrate of *M. tuberculosis* H37Rv, using specific antisera raised in rabbits. PI: assessment of the pre-immune serum showing no recognition of any mycobacterial protein. Molecular weight marker is shown on the left (P7708S ColorPlus Prestained Protein Marker, New England Biolabs) and the molecular weight observed for Rv0418 is shown to the right. (B) IEM assessment of the presence of Rv0418 on the surface of intact *M. tuberculosis* H37Rv bacilli (magnification: 40,000×). Proteins detected by anti-rabbit antibody conjugated to 10-nm colloidal gold particles are indicated by the black arrows. (C) Pre-immune serum showed no recognition of any mycobacterial proteins. The results showed detection of Rv0418 in TPS and surface of *M. tuberculosis* H37Rv.

**Figure 6 pcbi-1000824-g006:**
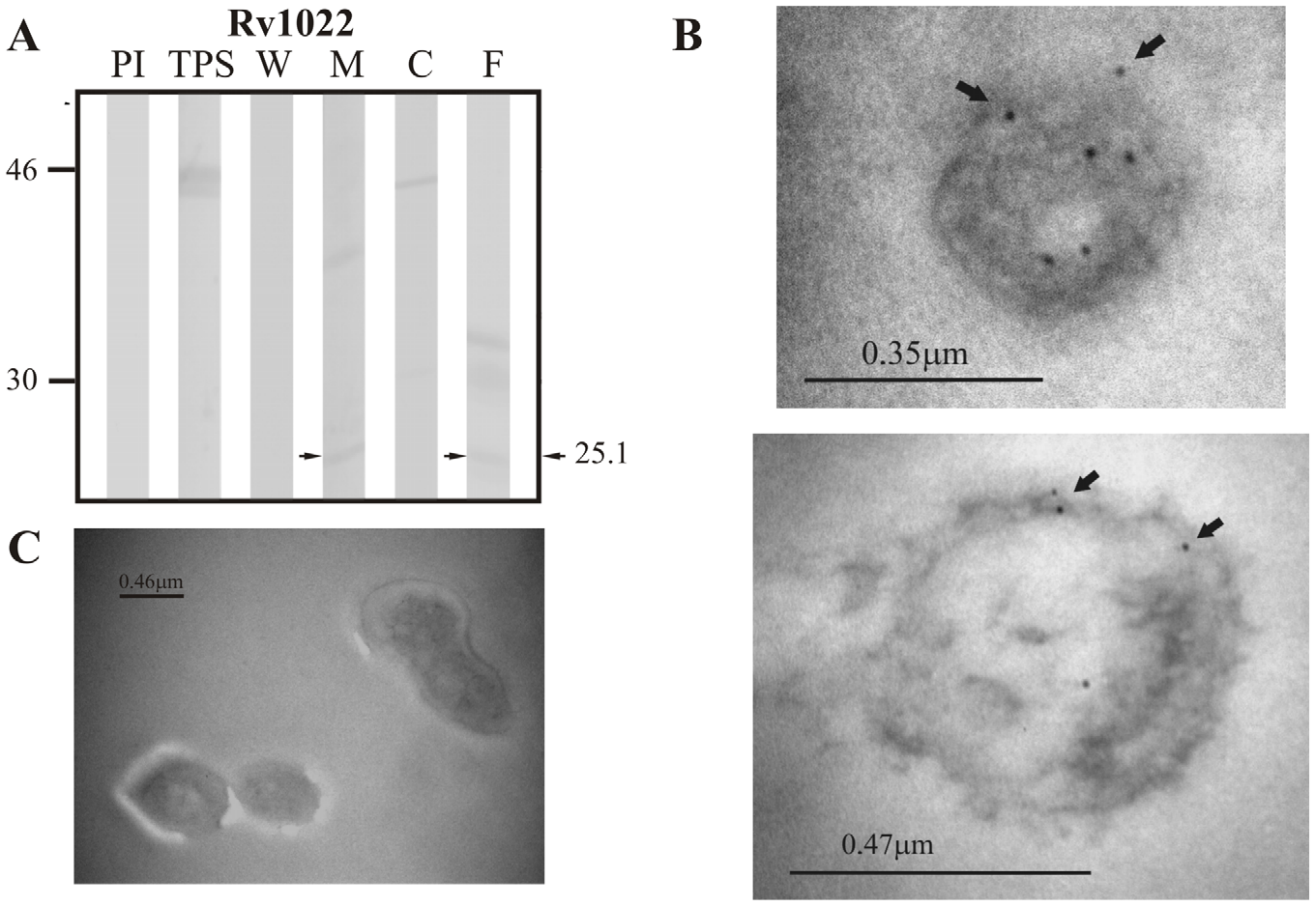
Experimental assessment of the subcellular localization of the candidate protein Rv1022 predicted as a surface/secreted by LipoP 1.0. (A) Immunoblotting assessment of the presence of Rv1022 in TPS: total protein sonicate, W: cell wall, M: membrane, C: cytosol and F: culture filtrate of *M. tuberculosis* H37Rv, using specific antisera raised in rabbits. PI: assessment of the pre-immune serum showing no recognition of any mycobacterial protein. Molecular weight marker is shown on the left (P7709S ColorPlus Prestained Protein Marker, New England Biolabs) and the molecular weight observed for Rv1022 is shown to the right. (B) IEM assessment of the presence of Rv1022 on the surface of intact *M. tuberculosis* H37Rv bacilli (magnification: 40,000×). Proteins detected by anti-rabbit antibody conjugated to 10-nm colloidal gold particles are indicated by the black arrows. (C) Pre-immune serum showed no recognition of any mycobacterial proteins. The results showed detection of Rv1022 in membrane, culture filtrate and surface of *M. tuberculosis* H37Rv.

**Figure 7 pcbi-1000824-g007:**
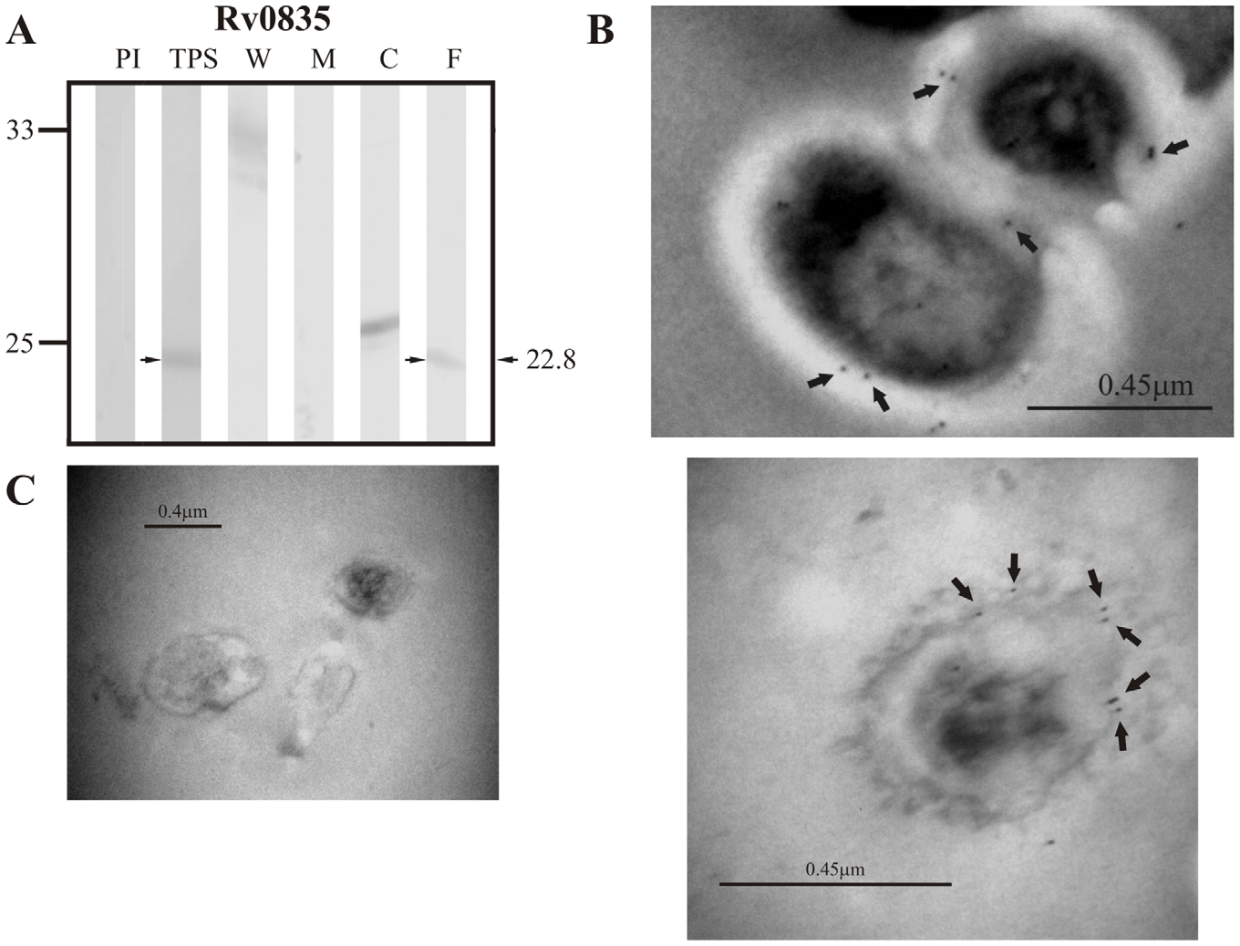
Experimental assessment of the subcellular localization of the candidate protein Rv0835 predicted as a surface/secreted by LipoP 1.0. (A) Immunoblotting assessment of the presence of Rv0835 in TPS: total protein sonicate, W: cell wall, M: membrane, C: cytosol and F: culture filtrate of *M. tuberculosis* H37Rv, using specific antisera raised in rabbits. PI: assessment of the pre-immune serum showing no recognition of any mycobacterial protein. Molecular weight marker is shown on the left (P7708S ColorPlus Prestained Protein Marker, New England Biolabs) and the molecular weight observed for Rv0835 is shown to the right. (B) IEM assessment of the presence of Rv0835 on the surface of intact *M. tuberculosis* H37Rv bacilli (magnification: 40,000×). Proteins detected by anti-rabbit antibody conjugated to 10-nm colloidal gold particles are indicated by the black arrows. (C) Pre-immune serum showed no recognition of any mycobacterial proteins. The results showed detection of Rv0835 in TPS, culture filtrate and surface of *M. tuberculosis* H37Rv.

Of the group of lipoproteins predicted by LipoP 1.0, the positive control Rv0418 was only detected in TPS at ∼51.9 kDa, which is close to the theoretical weight of 52.0 kDa expected for this protein (Figure 5A). Sera raised against the candidate lipoprotein Rv1022 did not detect this protein in TPS at the expected molecular weight of 25.8 kDa; however, it did recognize a band of ∼25.12 kDa in membrane fraction and culture filtrate (Figure 6A).Sera raised against the other candidate lipoprotein Rv0835 recognized a band of ∼22.8 kDa (expected molecular weight: 22.9 kDa) in TPS and culture filtrate (Figure 7A).

**Figure 8 pcbi-1000824-g008:**
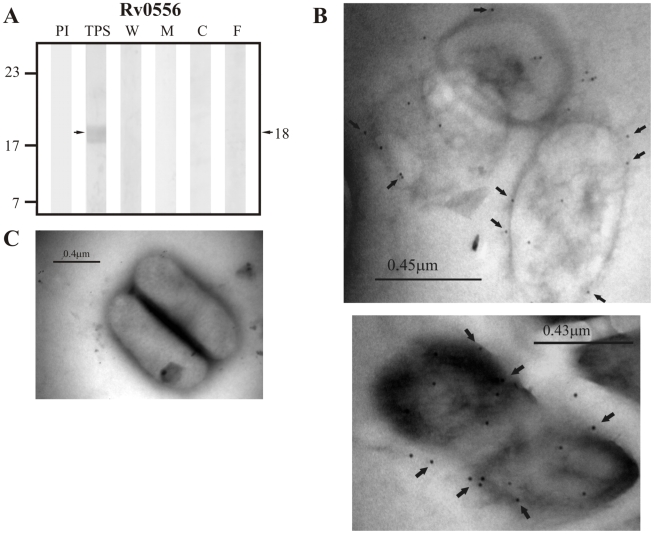
Experimental assessment of the subcellular localization of Rv0556, which was selected as positive control protein for surface/secreted predictions of SecretomeP 2.0. (A) Immunoblotting assessment of the presence of Rv0556 in TPS: total protein sonicate, W: cell wall, M: membrane, C: cytosol and F: culture filtrate of *M. tuberculosis* H37Rv, using specific antisera raised in rabbits. PI: assessment of the pre-immune serum showing no recognition of any mycobacterial protein. Molecular weight marker is shown on the left (P7709S ColorPlus Prestained Protein Marker, New England Biolabs) and the molecular weight observed for Rv0556 is shown to the right. (B) IEM assessment of the presence of Rv0556 on the surface of intact *M. tuberculosis* H37Rv bacilli (magnification: 40,000×). Proteins detected by anti-rabbit antibody conjugated to 10-nm colloidal gold particles are indicated by the black arrows. (C) Pre-immune serum showed no recognition of any mycobacterial proteins. The results showed detection of Rv0556 in TPS and surface of *M. tuberculosis* H37Rv.

**Figure 9 pcbi-1000824-g009:**
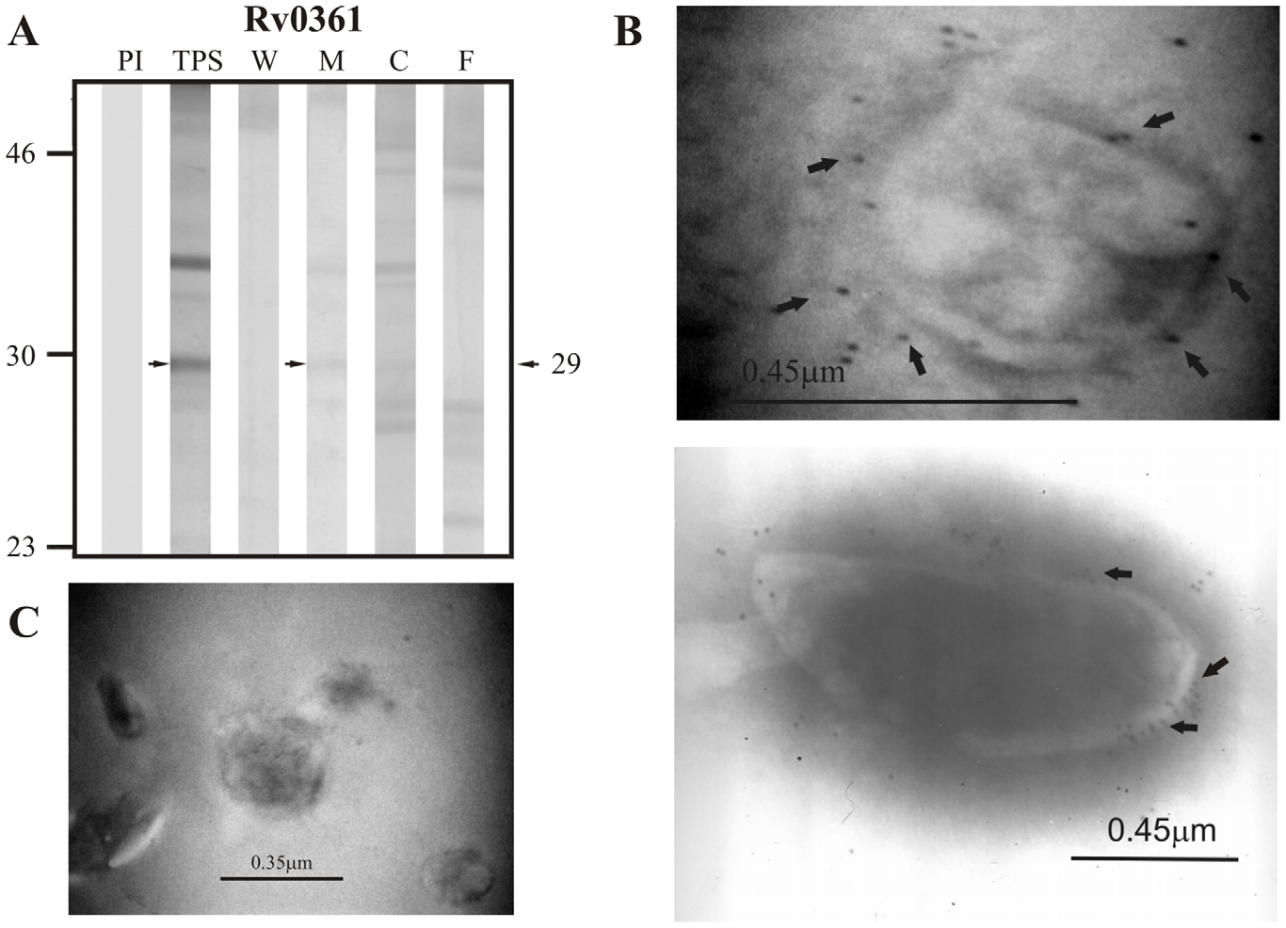
Experimental assessment of the subcellular localization of the candidate protein Rv0361 predicted as a surface/secreted by SecretomeP 2.0. (A) Immunoblotting assessment of the presence of Rv0361 in TPS: total protein sonicate, W: cell wall, M: membrane, C: cytosol and F: culture filtrate of *M. tuberculosis* H37Rv, using specific antisera raised in rabbits. PI: assessment of the pre-immune serum showing no recognition of any mycobacterial protein. Molecular weight marker is shown on the left (P7709S ColorPlus Prestained Protein Marker, New England Biolabs) and the molecular weight observed for Rv0361 is shown to the right. (B) IEM assessment of the presence of Rv0361 on the surface of intact *M. tuberculosis* H37Rv bacilli (magnification: 40,000×). Proteins detected by anti-rabbit antibody conjugated to 10-nm colloidal gold particles are indicated by the black arrows. (C) Pre-immune serum showed no recognition of any mycobacterial proteins. The results showed detection of Rv0361 in TPS, membrane and surface of *M. tuberculosis* H37Rv.

**Figure 10 pcbi-1000824-g010:**
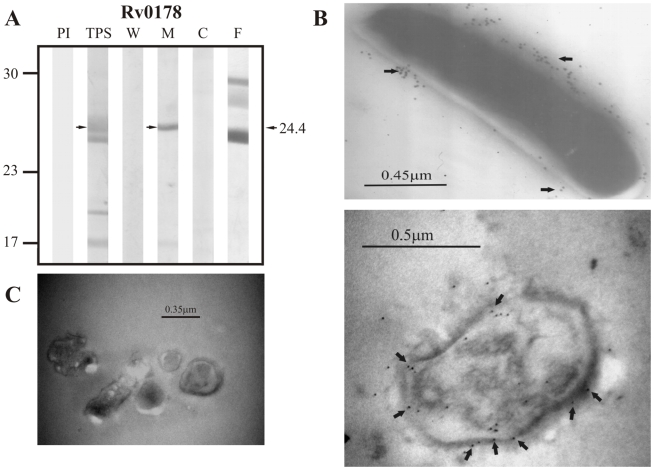
Experimental assessment of the subcellular localization of the candidate protein Rv0178 predicted as a surface/secreted by SecretomeP 2.0. (A) Immunoblotting assessment of the presence of Rv0178 in TPS: total protein sonicate, W: cell wall, M: membrane, C: cytosol and F: culture filtrate of *M. tuberculosis* H37Rv, using specific antisera raised in rabbits. PI: assessment of the pre-immune serum showing no recognition of any mycobacterial protein. Molecular weight marker is shown on the left (P7709S ColorPlus Prestained Protein Marker, New England Biolabs) and the molecular weight observed for Rv0178 is shown to the right. (B) IEM assessment of the presence of Rv0178 on the surface of intact *M. tuberculosis* H37Rv bacilli (magnification: 40,000×). Proteins detected by anti-rabbit antibody conjugated to 10-nm colloidal gold particles are indicated by the black arrows. (C) Pre-immune serum showed no recognition of any mycobacterial proteins. The results showed detection of Rv0178 in TPS, membrane and surface of *M. tuberculosis* H37Rv.

The results obtained for the group of non-classically secreted proteins predicted by SecretomeP 2.0 showed detection of the positive control Rv0556 in TPS at ∼18.0 kDa (theoretical molecular weight: 18.8 kDa) but not in culture filtrate nor in any of the subcellular fractions (Figure 8A).Sera raised against the candidate protein Rv0361, which has an expected molecular weight of 30.0 kDa, detected a band of ∼29.0 kDa in TPS and in the membrane fraction, but also detected other bands in culture filtrate and the cytoplasmic fraction at molecular weights different from the one expected for this protein (Figure 9A). Similarly, Rv0178, the other candidate protein of non-classical secretion was detected in TPS and in the membrane fraction at ∼24.4 kDa, which is close to the molecular weight of 25.9 kDa estimated for this protein. Other bands with molecular weights different from the one expected for this protein were also detected in culture filtrate (Figure 10A).

**Figure 11:**
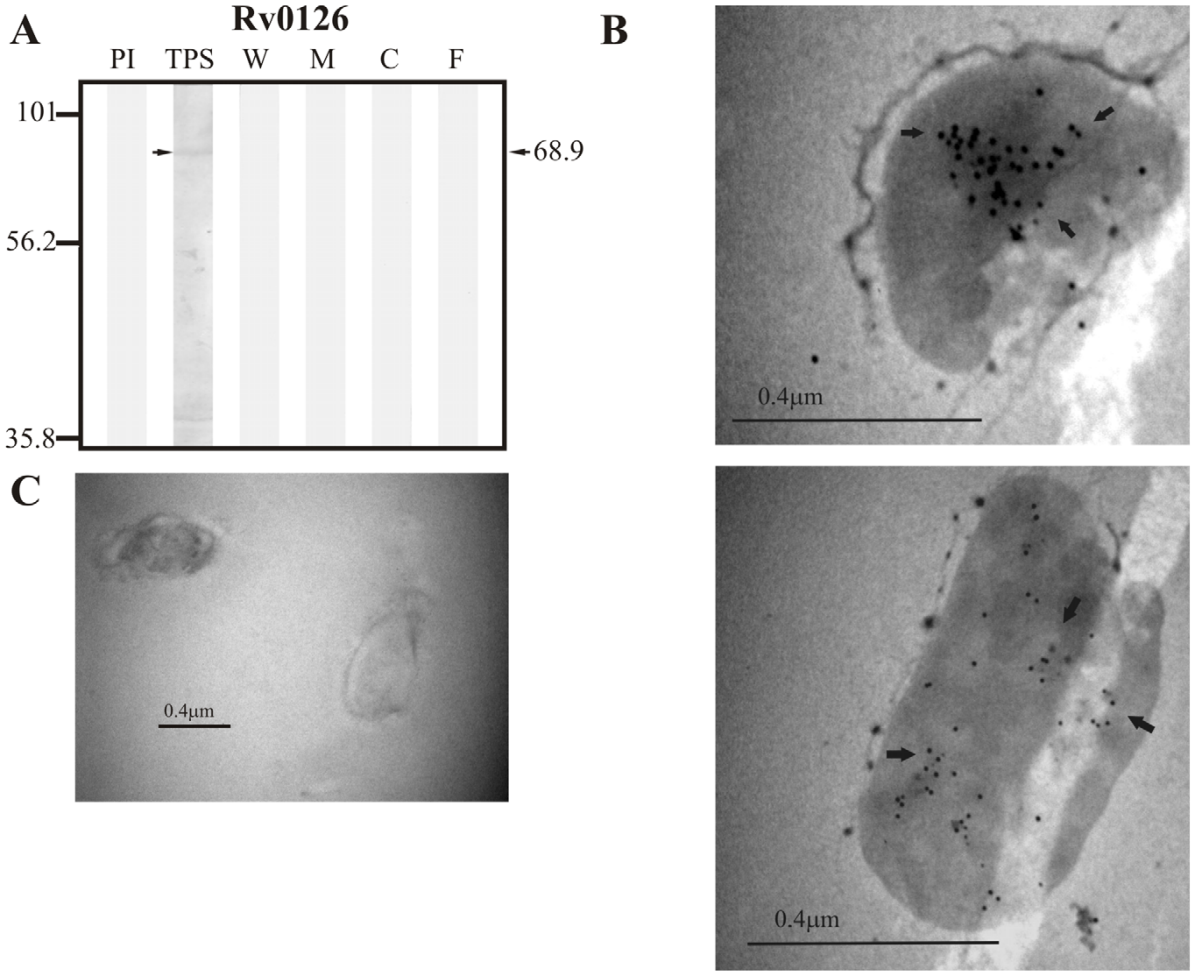
Experimental assessment of the subcellular localization of the negative control protein Rv0126. (A) Immunoblotting assessment of the presence of Rv0126 in TPS: total protein sonicate, W: cell wall, M: membrane, C: cytosol and F: culture filtrate of *M. tuberculosis* H37Rv, using specific antisera raised in rabbits. PI: assessment of the pre-immune serum showing no recognition of any mycobacterial protein. Molecular weight marker is shown on the left (P7708S ColorPlus Prestained Protein Marker, New England Biolabs) and the molecular weight observed for Rv0126 is shown to the right. (B) IEM assessment of the presence of Rv0126 in the cytoplasm of intact *M. tuberculosis* H37Rv bacilli (magnification: 40,000×). Proteins detected by anti-rabbit antibody conjugated to 10-nm colloidal gold particles are indicated by the black arrows. (C) Pre-immune serum showed no recognition of any mycobacterial proteins. The results showed detection of Rv0126 in TPS and the cytoplasm of *M. tuberculosis* H37Rv.

Finally, sera raised against the negative control protein Rv0126 (68.6 kDa) detected a band of ∼68.9 kDa in TPS (Figure 11A), whereas no proteins were recognized in assays by sera raised against the negative control protein Rv1326c (81.7 kDa) (data not shown).

**Figure 12:**
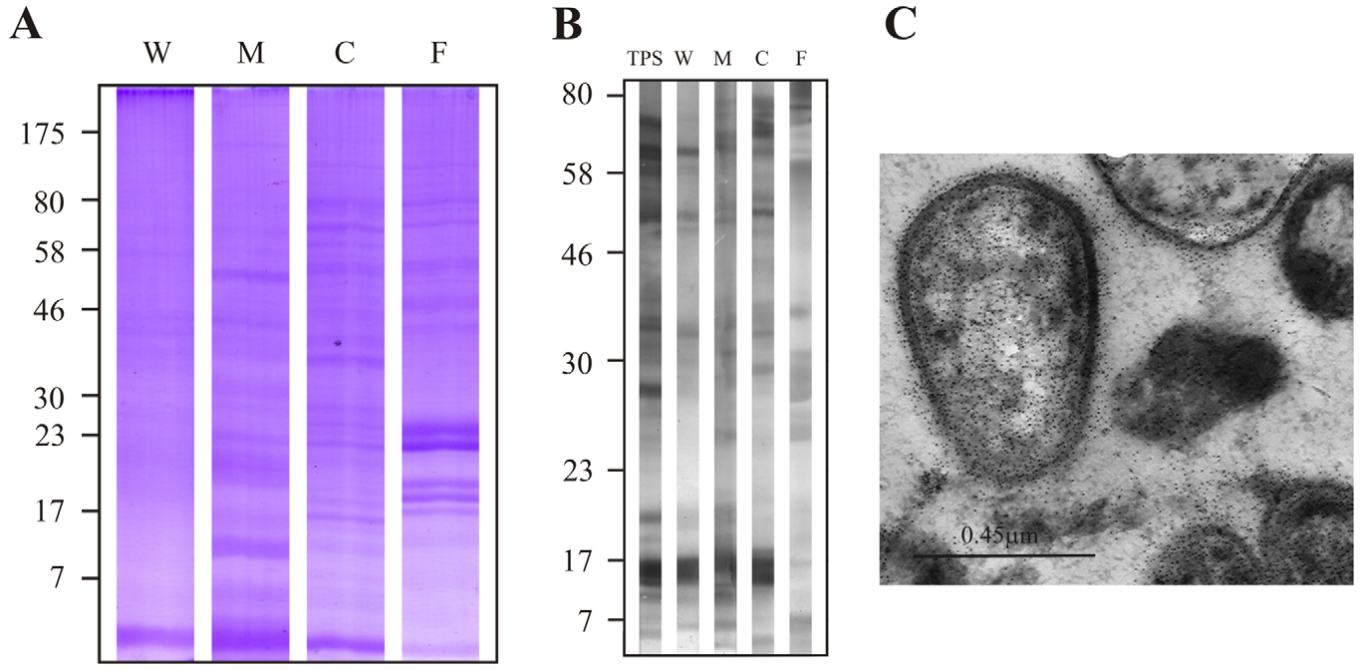
Positive control of immunoblotting assays. (A) Colloidal Coomassie Blue Staining of SDS-gel containing W: cell wall, M: membrane, C: cytosol fractions and F: culture filtrate of *M. tuberculosis* H37Rv. (B) Recognition of several protein bands of strong intensity by sera raised against a crude protein sonicate of *M. tuberculosis* H37Rv (TPS) and in all subcellular fractions and the culture filtrate. (C) Positive control of IEM studies: Serum raised against crude sonicate of *M. tuberculosis* H37Rv (TPS) recognized proteins in all subcellular compartments (magnification: 40,000×).

In addition, the subcellular fractions were assessed with Colloidal Coomassie Blue Staining of an SDS-gel and sera raised against crude sonicate of *M. tuberculosis* H37Rv by Western blot. The results of Colloidal Coomassie Blue gel staining showed numerous proteins with different migration distances in the different fractions and in the culture filtrate (Figure 12A). As observed in Figure 12B, several protein bands of strong intensity were recognized in all subcellular fractions and culture filtrate, as well as in TPS by sera raised against a total sonicate of *M. tuberculosis*, therefore indicating that antibody and cell fraction concentrations were appropriate. None of the pre-immune sera recognized proteins in TPS (pre-immune lanes in Figures 1–11), thus indicating that antisera were specific against their corresponding mycobacterial antigens.

The same sera raised against B cell epitopes of the three groups of proteins (candidate, positive and negative control proteins) were used in IEM experiments to determine the localization of the candidate proteins and assess whether the localization of the positive and negative controls agreed with their reported localizations. It should be noted that IEM studies were not carried out for Rv1733c, Rv3069 and Rv1326c due to the negative reactivity shown by their antisera, which is probably indicating that peptides used for immunization corresponded to conserved immunologically silent sequences. The results showed colloidal gold particles of about 10 nm in the surface of intact *M. tuberculosis* H37Rv bacilli when assessing sera raised against Rv0200 (Figure 1B), Rv0403c (Figure 2B), Rv1280c (Figure 3B), Rv3630 (Figure 4B), Rv0418 (Figure 5B), Rv1022 (Figure 6B), Rv0835 (Figure 7B), Rv0556 (Figure 8B), Rv0361 (Figure 9B), and Rv0178 (Figure 10B), whereas colloidal gold particles were observed only in the cytoplasm with sera raised against the cytoplasmic protein Rv0126 (Figure 11B). Pre-immune sera showed no recognition of any mycobacterial protein (panel C in Figures 1–11), and sera raised against total *M. tuberculosis* H37Rv protein sonicate recognized proteins in all subcellular compartments (Figure 12C). These IEM results are consistent with the subcellular localizations predicted for these proteins in the computational phase (Table 2).

## Discussion

The use of bioinformatics tools and web-available databases has facilitated the identification of proteins that are expressed on the cell surface or secreted to the extracellular milieu of pathogenic organisms by searching for intrinsic secretion signals and membrane anchoring features on the sequences of hypothetical proteins encoded in their genomes. Although the different computational methods currently available can be used to predict the subcellular localization of a given protein, their use has to be first validated by experimentally determining the accuracy of their predictions.

The large number of proteins predicted independently by SecretomeP 2.0 in this study could be explained by the capacity of this tool to identify all proteins that are secreted but do not contain a signal sequence (probability of signal sequence absence), which would include proteins that are secreted through unknown secretion mechanisms. Even though SecretomeP 2.0 is considered a feature-based tool, its predictions are not based on the identification of specific sequence feature or motif, as occurs with Signal 3.0, TatP 1.0 and LipoP 1.0, and therefore this tool can identify proteins independently of their secretory mechanism [29].

The experimental phase provided evidence of secretion to the mycobacterial surface for six candidate proteins. In the case of proteins predicted to be secreted via the Sec-dependent pathway, the candidate protein Rv0403c contains two transmembrane helices according to TMHMM 2.0, the first of which is located between amino acids 7 and 29 and overlaps with the signal sequence predicted by both SignalP 3.0 and Phobius between amino acids 1 and 33 (Table 2). Rv0403c was detected in TPS as well as in the membrane fraction and was observed in the surface of intact bacilli by IEM (Figure 2A and B). All these results are is consistent with the predictions of the Machine Learning tools and would indicate that this protein is anchored to mycobacterial surface and involved in cell wall and cell processes, as appears annotated in TubercuList.

Rv3630, which was predicted in the computational phase to be secreted via the Tat secretory machinery, is also annotated in TubercuList as a probable conserved integral membrane protein involved in cell wall and cell processes. This protein is predicted to contain a signal sequence between amino acids 1 and 40 (according to TatP 1.0), and to have 11 transmembrane helices (according to TMHMM 2.0), whereas Phobius predicted 12 transmembrane helices and no signal sequence (Table 2). These predictions of transmembrane helices are in agreement with this protein's annotation as well as with the predictions of localization n the plasma membrane by the general localization tools. This protein was detected in TPS and the surface of intact bacilli by rabbit sera but was not recognized in any of the subcellular fractions nor in culture filtrate (Figure 4A and B), probably due to its low abundance in these fractions, as suggested by its weak reactivity in immunoblotting analyses and the few gold particles observed in IEM studies, or perhaps due to the low immunogenicity of the chemically synthesized peptides used to detect its presence.

In Rv1022, which was predicted by LipoP 1.0, there is no evidence of transmembrane helices since Phobius shows a signal sequence in the same region predicted to be a transmembrane helix by TMHMM 2.0 (Table 2). General subcellular localizers indicate that this protein is intracytoplasmic, which is not consistent with the known subcellular localization of lipoproteins because even though lipoproteins do not contain transmembrane helices, it seems that the lipobox motif and the Lgt and Lsp cleavage sites are necessary and sufficient to ensure their anchorage to the membrane [16], [49]. These computational predictions, together with the detection of Rv1022 in culture filtrate (Figure 6A) and on the surface of intact bacilli (Figure 6B) indicate that secretion of this protein is driven by the signal sequence (nevertheless, gold particles might also correspond to any of the other proteins that were detected in immunoblotting assays), while the lack of transmembrane helices might indicate that it is secreted to the extracellular milieu and is consistent with the lack of any of the residues in position +2 known to be relevant for membrane anchorage [50], [51]. The presence of this protein both in membrane and culture filtrate could be explained by the gradual liberation of proteins during mycobacterial growth in culture, but it is intriguing that the protein was not also detected in the cell wall fraction considering that mycobacterial secreted proteins are usually expressed in large amounts during the first days of culture and can be found in trace amounts inside the bacillus [52]. Regarding Rv0835, the predictions indicated that it contains a signal sequence (which was also predicted by Phobius), is not anchored to membrane and has no transmembrane regions. Interestingly, Gpos-PLoc and PSORTb v.2.0.4 located this protein in the cytoplasm, whereas PA-SUB v.2.5 located it in the plasma membrane (Table 2). In the experimental phase, this protein was detected in TPS and culture filtrate (Figure 7A), which provides additional evidence that this hypothetical lipoprotein is exported to the extracellular milieu, as reported by Malen *et al.* [41]. This protein is similar to the positive control protein Rv0418 in that it has been reported as a secreted/surface protein by proteomics studies and both are hypothetical lipoproteins; however, the positive control was identified both in the membrane and the culture filtrate by Gu *et al.* [37] and Mawuenyega *et al.* [53], in contrast to the candidate protein Rv0835 which has been only reported in the culture filtrate [41].

Regarding non-classical secretion, important mycobacterial proteins such as ESAT-6 (Rv3875) and CFP-10 (Rv3874) have been detected in culture filtrate even though they do not seem to contain a signal sequence in their N-terminal regions [54]. Both of these proteins were positively identified by SecretomeP 2.0 in the computational phase (Table S4), therefore giving additional support to the use of this tool in our study.

The other two protein candidates, Rv0361 and Rv0178, chosen from the set of secreted/surface proteins predicted by SecretomeP 2.0, have no predicted signal peptides according to the other feature-based tools (Table 2), which indicates that they are not secreted to the mycobacterial surface via the classical pathway but rather through yet unknown mechanisms. Both proteins were identified in TPS, in the membrane fraction as well as in the bacillus surface (Figures 9 and 10); however, sera raised against Rv0178 also recognized protein bands of lower molecular weight in immunoblotting assays, thus suggesting a potential cleavage of this protein.

According to the computational analysis, Rv0178 has a transmembrane helix, as predicted by Phobius, and is located in the extracellular compartment, according to Gpos-PLoc. Previous studies suggest that the mycobacterial *mce1* operon, which contains the gene encoding Rv0178, plays an important role in mycobacterial invasion and persistent infection in mice, as well as a possible role in mycobacterial virulence [55], [56].

The detection of intense bands at the same molecular weight in immunoblotting analyses of TPS suggests that subcellular fractions were not completely pure (Figure 12B), which could explain why subcellular fractionation results for positive and negative control proteins (e.g. Rv0126) were not in some cases consistent with their known localizations; nevertheless, rabbit sera did recognize these proteins in their expected localization in IEM studies (surface or cytoplasm). For this reason, IEM results were considered more reliable than the ones obtained in immunoblotting assays with subcellular fractions when analyzing the localization of each candidate protein.

On the other hand, the absence of recognition of Rv1326c, Rv1733c and Rv3069 by rabbit antisera suggests that epitopes were probably not strong enough as to induce a good antibody response and therefore obtain large amounts of antibodies for immunoblotting and IEM assays, or alternatively, that the peptide sequences chosen in this study were immunologically silent, as occurs with malarial high activity binding peptides.

Predicting protein regions that are likely to be exposed on the protein surface and therefore accessible to interaction with host immune system molecules (humoral or cellular) is of key importance both in the design of vaccine components and the development of immunodiagnostic methods. Linear B cell epitopes predicted and synthesized in this study corresponded to continuous epitopes that were selected by using classical sequence prediction methodologies (based on the physicochemical profile of each amino acid). Nevertheless, even though these tools are continuously being improved, there are still limitations in their use [39], [40], [57].

In conclusion, the accuracy of the subcellular localization predictions yielded by different computational tools was confirmed by the experimental evidence gathered in this study showing experimental confirmation on the secretion of Rv0403c, Rv3630 and Rv1022 for the first time. Moreover, their possible secretory mechanism is suggested based on the tool that predicted their secretion (SignalP 3.0, TatP 1.0 and LipoP 1.0, respectively). The results indicate that secreted/surface proteins can be pre-identified based on thorough decision-making protocols supported by statistical learning, which would optimize the identification of new drug and vaccine targets and could be extrapolated to the identification of other protein sets, either in the same or in other biological agents.

Even though the mycobacterial cell envelope is a complex structure, it is important to highlight that the Machine Learning methods followed in this study are a good approximation to the problem of identifying surface proteins, which compared to other methodological approaches such as proteomics is a good strategy for narrowing the search to a given set of proteins before conducting expensive and time-consuming experimental studies.

## Methods

### Ethics statement

All rabbits used in this study were taken care according to procedures established by the Office for Protection from Research Risks (OPRR, Department of Health and Human Services, USA).

### Computational phase: retrieval and analysis of the *M. tuberculosis* H37Rv genome

Protein sequences from the *M. tuberculosis* H37Rv genome were retrieved from two databases: (1) TubercuList, available at http://genolist.pasteur.fr/TubercuList/ and (2) Sanger Institute, available at ftp://ftp.sanger.ac.uk/pub/tb/sequences/TB.pep. Protein sequences downloaded from these databases were exhaustively compared in order to obtain depurated protein sets.

### Feature-based subcellular localization predictions (specific approach)

Protein sequences were screened using feature-based tools to identify signal sequences associated to each of the secretory pathways (Sec, Tat and lipobox), taking care of selecting the option Gram-positive bacteria. Prediction of signal sequences for secretion via the classical pathway (Sec-dependent) was carried out using the SignalP 3.0 server (http://www.cbs.dtu.dk/services/SignalP/). The threshold for this feature-based tool was set to a ≥0.5 score according to the HMM result. For secretion via the alternative Tat secretory pathway, we used the TatP 1.0 server (http://www.cbs.dtu.dk/services/TatP/) to search for Tat motifs between the *n* and *h* regions of the signal peptide sequence, considering a score of ≥0.5. Additionally, we used LipoP1.0 (http://www.cbs.dtu.dk/services/LipoP/) to predict lipoprotein lipobox motifs within the first 70 amino acids of each sequence. Proteins showing the best type II signal sequence score and a high probability of SPase II cleavage site were included in the analysis. SecretomeP 2.0 (http://www.cbs.dtu.dk/services/SecretomeP/) was used to identify proteins with no apparent signal sequence that might be secreted via other pathways, according to a selection threshold of ≥0.5.

Transmembrane topology in the proteins selected based on the predictions of the feature-based tools was predicted using TMHMM 2.0 (http://www.cbs.dtu.dk/services/TMHMM/). This tool allows identifying the presence, localization and orientation of transmembrane helices. Taking into account the number of amino acids expected in a helix (ExpAA), a protein is likely to cross the membrane or have a signal sequence if this number is larger than 18; if the helix lies within the first 60 residues (first 60 amino acids), the protein might not have a transmembrane helix but could instead have a signal sequence.

Phobius (http://phobius.sbc.su.se/) was used in order to distinguish between a signal sequence (either for secretion via classical or alternative pathways) and an N-terminal transmembrane helix, by identifying possible α-helical conformations in the *h* region of the signal sequence that could be mistakenly classified as a transmembrane region [36].

### General subcellular localization predictions (general approach)

Proteins selected based on the predictions of the feature-based tools were further analyzed with general subcellular localization classifiers using the following bioinformatics predictors: Gpos-PLoc (http://www.csbio.sjtu.edu.cn/bioinf/Gpos/), PSORTb v.2.0.4 (http://www.psort.org/psortb/) and PA-SUB v.2.5 (http://pasub.cs.ualberta.ca:8080/pa/Subcellular). In all cases, predictions were performed selecting the option Gram-positive bacteria.

### Selection of candidate proteins

Proteins predicted to have a signal sequence for secretion via a specific secretory pathway and the ones identified by SecretomeP 2.0 were compared to the set of 100 membrane proteins reported by Gu *et al.* [37] and Sinha *et al.* [58]. Surface proteins derived from this comparison and preferably exclusive for each secretory pathway were selected as positive controls, taking care of selecting those that had at least one published study confirming their secretion or surface localization. On the other hand, negative controls were selected from the list of cytoplasmic proteins reported in the TBsgc database (http://www.doe-mbi.ucla.edu/TB/) and were analyzed using the same prediction methodology applied to candidate proteins and positive controls to confirm the absence of a signal sequence, localization in a subcellular compartment different from the surface or extracellular milieu, and the lack of transmembrane helices.

Likewise, proteins in the *M. tuberculosis* H37Rv genome predicted to be secreted preferably via a single pathway were chosen as candidate proteins in the experimental phase. These proteins were selected based on a high signal sequence probability score, as mentioned above. Preferably, only proteins that had no previous publications were selected.

Furthermore, proteins selected on the basis of the above mentioned criteria had to be predicted to be located on the cell surface by any of the general subcellular localization tools, especially Gpos-PLoc and PSORTb v. 2.0.4 (algorithms validated for prediction in mycobacterial proteins that have high sensitivity and specificity). Additionally, candidate proteins had to contain at least one transmembrane helix, except in the case of lipoproteins, which are anchored to the cell surface via the lipobox motif.

In summary, each secretory pathway included a protein whose membrane localization had been confirmed by proteomics and two additional proteins obtained from the prediction analysis carried out over the entire genome.

### Prediction of linear B cell epitopes

Linear B cell epitopes were predicted using AntheProt, which is available at http://antheprot-pbil.ibcp.fr/ and the BepiPred 1.0 server available at http://www.cbs.dtu.dk/services/BepiPred/. Linear B cell epitopes predicted by BepiPred were compared to the values given for each amino acid by AntheProt regarding three physicochemical profiles: combined antigenicity, hydrophilicity and solvent accessibility. The values obtained for each profile were averaged in groups of 20 amino acids. For each protein, regions having the best averages were considered as good linear B cell epitopes and their selection involved also their exclusive presence in *M. tuberculosis*, as indicated by homology comparison using the BLASTp tool.

### Experimental phase: peptide synthesis

Two 20-amino-acid-long, non-overlapping peptides selected based on the linear B cell epitope predictions performed for each protein were chemically synthesized by the multiple solid phase synthesis methodology [59], using BHA resin (0.7 meq/mg) and t-*boc* protected amino acids. Peptides were freeze-dried, purified by reverse phase high performance liquid chromatography (RP-HPLC) and characterized by MALDI-TOF mass spectrometry (Bruker, USA). Cysteine and glycine residues were added at both ends and oxidization was carried out at pH 7.5 to enable their polymerization.

### Rabbit immunization

Two animals per peptide previously determined to be nonreactive to *M. tuberculosis* sonicate were subcutaneously immunized with each peptide. Each animal received a mixture of 0.6 mg/ml of polymerized synthetic peptide emulsified in Freund's incomplete adjuvant on days 0, 20 and 40. Sera were collected before the first immunization (pre-immune sera) and 20 days after the third immunization (immune sera). Proteins for which sera were already available at the FIDIC's serum library, as well as those for which peptides were synthesized for this study, are shown in Table 3.

### *M. tuberculosis*, *M. smegmatis* and *E. coli* cultures and sonicates

*M. tuberculosis* H37Rv (ATCC 27294) and *Mycobacterium smegmatis* (Paris 4995) were grown in Middlebrook 7H9 Broth with 0.05% Tween 80 enriched with 10% OADC (Oleic Acid, Albumin, Dextrose and Catalase) and 0.2% Glycerol for 15 days to mid-log phase of growth at 37°C and 33°C, respectively [60]. Bacteria were harvested by centrifugation at 12,500 *g* for 30 min, suspended in 1× phosphate buffered saline (PBS) and stored at −20°C. For *E. coli* TOP10 (Invitrogen, CA, USA), 4 ml of Luria Bertani (LB) sterile medium were inoculated with the bacteria and cultured for 16–18 h at 37°C under constant shaking.

Ten grams (wet weight) of mycobacteria and *E. coli* were suspended in 20 ml of 1× PBS containing DNase, RNase and proteinase inhibitor cocktail (1 mM phenylmethylsulfonylfluoride [PMSF], 1 mM EDTA, 1 mg/ml of leupeptin and 1 mg/ml of Pepstatin A). Cells were sonicated for 20 min using a Branson 450 Sonifier ultrasonic cell disruptor, setting the amplitude to 4 and the duty cycle to 90%. The sonicate was centrifuged at 650 *g* for 20 min, skimmed off and the supernatant was then centrifuged at 36,000 *g* for 45 min at 4°C. Protein concentration was determined by the Bicinchoninic Acid (BCA) method [61] using bovine serum albumin (BSA) as standard. This *M. tuberculosis* H37Rv total protein sonicate (TPS) was used in the all immunodetection assays.

*E. coli* and *M. smegmatis* sonicates were individually coupled to CNBr-activated Sepharose 4B (GE Healthcare Life Sciences, NJ, USA), according to the manufacturer's recommendations. Rabbit antisera (pre-immune and immune) were pre-adsorbed on *E. coli*-Sepharose and *M. smegmatis*-Sepharose affinity columns to remove crossreactive antigens. Briefly, 5 ml of each serum was added to 4 ml of sonicate-Sepharose affinity columns and left in a gentle rotating/shaking mode for 20 min at room temperature. This procedure was done twice using a new sonicate-Sepharose affinity column each time.

### Separation of culture filtrate and subcellular fractions

*M. tuberculosis* H37Rv culture aliquots of 25 ml were centrifuged at 10,000 *g* for 20 min at 4°C and the supernatant was filtered under sterile conditions using a 0.2-µm pore membrane to obtain the culture filtrate. To obtain the subcellular fractions, the pellet was washed thrice with 1× PBS, resuspended in proteinase inhibitor cocktail, sonicated for 15 min on an ice bath (work cycle: 80%, amplitude: 3), let to settle for 15 min at 4°C and again sonicated same as before. This procedure was carried in two stages to avoid sample overheating and thus maintain protein integrity.

This sonicate was centrifuged at 3,000 *g* for 5 min at 4°C, the pellet (unbroken cells) was discarded and the supernatant was centrifuged at 27,000 *g* for 1 h at 4°C. The pellet was resuspended in lysis buffer containing lysozyme (without DNAse and RNAse) and centrifuged one more time for 1 h at 4°C and 27,000 *g*. The pellet from this centrifugation, which corresponded to the cellular wall, was resuspended in ammonium bicarbonate, while the supernatants from the first and second centrifugations were pooled and centrifuged at 100,000 *g* for 4 h at 4°C. The supernatant obtained from this centrifugation was labeled as cytosolic fraction and centrifuged for 4 h at 100,000 *g* and 4°C to remove traces of membrane proteins (pellet). All pellets were pooled and labeled as membrane fraction.

The cell wall, membrane, cytosolic and culture filtrate were washed with DNAse-RNAse free lysis buffer, poured into 3500 MWCO dialysis tubing (Spectra/Por, CA, USA) and dialyzed against 10 mM ammonium bicarbonate for 24 h at 4°C, changing the buffer thrice. Dialyzed fractions were concentrated with polyethylenglycol, quantified by BCA [62], [63] and stored at −70°C.

### Sodium dodecyl sulfate polyacrylamide gel electrophoresis (SDS-PAGE) and immunoblotting

Proteins from the *M. tuberculosis* TPS and subcellular fractions were separated in a discontinuous SDS-PAGE system using a 10–20% (*w*/*v*) acrylamide gradient and commercial molecular mass markers (New England Biolabs Inc. MA, USA and BIO-RAD, CA, USA) to estimate protein molecular weights. One milligram of each sample was loaded per gel and transferred to nitrocellulose membrane (Hybond 203c, Pharmacia). Membranes were blocked with 5% skimmed milk diluted in TBS–T (0.02 M Tris–HCl at pH 7.5, 0.05 M NaCl and 1% Tween 20), cut into strips and incubated with pre-adsorbed rabbit sera diluted 1∶100 in blocking solution. Strips were then incubated for 1 h with 1∶4,500 alkaline-phosphatase-conjugated anti-rabbit IgG antibody (Vector Laboratories, Inc. CA, USA), followed by five washes with TBS–T. The reaction was developed with NBT/BCIP kit (Promega, WI, USA), according to the manufacturer's recommendations.

Each membrane strip was incubated for 1 h at room temperature with a 1∶100 dilution of pre-immune or immune sera diluted in blocking solution. Anti-rabbit IgG antibody coupled to alkaline phosphatase (Vector Laboratories, Inc.) diluted 1∶5,000 in blocking solution was used as secondary antibody. Immunoreactivity was developed by a colorimetric enzymatic reaction (NBT/BCIP). Hyperimmune anti-*M. tuberculosis* TPS serum was used as positive control. Additionally, an SDS-gel containing culture filtrate proteins and all subcellular fractions was stained with Colloidal Coomassie Blue.

### Immunoelectron microscopy (IEM)

An inoculum of *M. tuberculosis* H37Rv was resuspended and fixed for 2 h using 4% paraformaldehyde/0.5% glutaraldehyde in 1× PBS and then dehydrated through 15 min immersions in a series of graded ethanol (50%, 70%, 90%, and 100% twice), centrifuging samples for 15 min at 2,500 *g* between immersions. Bacilli were gradually embedded and polymerized in LR-White resin (SPI supplies, PA, USA), using accelerator for cold polymerization as specified by the manufacturer (1µl accelerator/500 µl resin). Ultrathin sections (400 nm thick) were mounted on 300-mesh nickel grids coated with collodion support. For immunolabeling, grids were blocked with 5% BSA–0.1% Tween 20 for 30 min and subsequently washed carefully drop by drop with 0.5% BSA–0.1% Tween 20 in 1× PBS.

Grids were incubated overnight at 4°C with a 1∶20 dilution of primary antibody (either pre-immune or immune sera), washed thrice as before and immersed in a 1∶50 dilution of anti-rabbit antibody coupled to 10-nm colloidal gold particles for 1 h at room temperature [64]. For each protein, rabbits' pre-immune sera were used as negative control and sera raised against *M. tuberculosis* TPS were used as positive control, respectively. Finally, grids were stained with 6% uranyl acetate to enhance image contrast, then washed with distilled water and dried before being examined in a Morgani 268 digital transmission electron microscopy.

**Figure 13:**
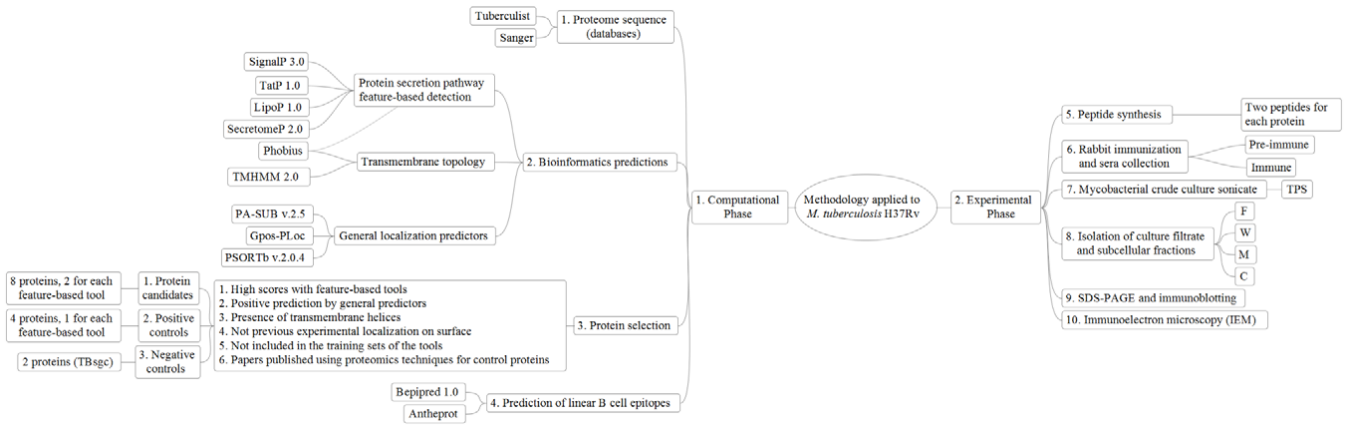
Flow chart of the methodology. The flow chart describes the different steps followed in the computational and the experimental phases. TPS: total protein sonicate, W: cell wall, M: membrane, C: cytosol and F: culture filtrate of *M. tuberculosis* H37Rv.

Figure 13 summarizes all steps followed in the computational phase as well as in the experimental phase.

## Supporting Information

Table S1 Proteins predicted by SignalP 3.0. This file contains the predictions yielded by SignalP 3.0 for the complete genome of *M. tuberculosis* H37Rv with their corresponding transmembrane topology and general localization predictions. In the last table column entitled “shared”, proteins that were also positively predicted by any of the other feature-based tools are labeled with an “x” (File format: .xls).
(0.33 MB XLS)

Table S2 Proteins predicted by TatP 1.0. This file contains the predictions yielded by TatP 1.0 on the complete genome of *M. tuberculosis* H37Rv with their corresponding transmembrane topology and general localization predictions. In the last table column entitled “shared”, proteins that were also positively predicted by any of the other feature-based tools are labeled with an “x” (File format: .xls).
(0.12 MB XLS)

Table S3 Proteins predicted by LipoP 1.0. This file contains the predictions yielded by LipoP 1.0 on the complete genome of *M. tuberculosis* H37Rv with their corresponding transmembrane topology and general localization predictions. In the last table column entitled “shared”, proteins that were also positively predicted by any of the other feature-based tools are labeled with an “x” (File format: .xls).
(0.05 MB XLS)

Table S4 Proteins predicted by SecretomeP 2.0. This file contains the predictions yielded by SecretomeP 2.0 on the complete genome of *M. tuberculosis* H37Rv with their corresponding transmembrane topology and general localization predictions. In the last table column entitled “shared”, proteins that were also positively predicted by any of the other feature-based tools are labeled with an “x” (File format: .xls).
(0.45 MB XLS)

Table S5 Computational prediction on the proteins identified experimentally by Gu et al. and Sinha et al. This file contains the predictions yielded by the feature-based tools for 100 proteins identified experimentally in the proteomics studies carried out by Gu et al. and Sinha et al. with their corresponding transmembrane topology and general localization predictions. In the last table column entitled “shared”, proteins that were also positively predicted by any of the other feature-based tools are labeled with the number “1” (File format: .xls).
(0.08 MB XLS)

Table S6 Negative controls. This file contains the predictions yielded by the machine-learning tools for the 9 cytoplasmic proteins reported in TBsgc (File format: .xls).
(0.03 MB XLS)

## References

[1] WHO (2009). Global tuberculosis control: surveillance, planning, financing, World Health Organization.

[2] Dietrich J, Doherty TM (2009). Interaction of *Mycobacterium tuberculosis* with the host: consequences for vaccine development.. APMIS.

[3] Sigler K, Hofer M (1997). Biotechnological aspects of membrane function.. Crit Rev Biotechnol.

[4] Smith I (2003). *Mycobacterium tuberculosis* pathogenesis and molecular determinants of virulence.. Clin Microbiol Rev.

[5] Cole ST, Brosch R, Parkhill J, Garnier T, Churcher C (1998). Deciphering the biology of *Mycobacterium tuberculosis* from the complete genome sequence.. Nature.

[6] Goulding CW, Apostol M, Anderson DH, Gill HS, Smith CV (2002). The TB Structural Genomics Consortium: Providing a Structural Foundation for Drug Discovery.. Current Drug Targets - Infectious Disorsers.

[7] Zhang LJ, Wang XE, Peng X, Wei YJ, Cao R (2006). Proteomic analysis of low-abundant integral plasma membrane proteins based on gels.. Cell Mol Life Sci.

[8] DiGiuseppe Champion PA, Cox JS (2007). Protein secretion systems in Mycobacteria.. Cell Microbiol.

[9] Sargent F, Berks BC, Palmer T (2006). Pathfinders and trailblazers: a prokaryotic targeting system for transport of folded proteins.. FEMS Microbiol Lett.

[10] Braunstein M, Espinosa BJ, Chan J, Belisle JT, Jacobs WR (2003). SecA2 functions in the secretion of superoxide dismutase A and in the virulence of *Mycobacterium tuberculosis*.. Mol Microbiol.

[11] Schatz G, Dobberstein B (1996). Common principles of protein translocation across membranes.. Science.

[12] Tjalsma H, Bolhuis A, Jongbloed JD, Bron S, van Dijl JM (2000). Signal peptide-dependent protein transport in *Bacillus subtilis*: a genome-based survey of the secretome.. Microbiol Mol Biol Rev.

[13] Rezwan M, Grau T, Tschumi A, Sander P (2007). Lipoprotein synthesis in mycobacteria.. Microbiology.

[14] Sander P, Rezwan M, Walker B, Rampini SK, Kroppenstedt RM (2004). Lipoprotein processing is required for virulence of *Mycobacterium tuberculosis*.. Mol Microbiol.

[15] Hirose I, Sano K, Shioda I, Kumano M, Nakamura K (2000). Proteome analysis of *Bacillus subtilis* extracellular proteins: a two-dimensional protein electrophoretic study.. Microbiology.

[16] Kamalakkannan S, Murugan V, Jagannadham MV, Nagaraj R, Sankaran K (2004). Bacterial lipid modification of proteins for novel protein engineering applications.. Protein Engineering, Design and Selection.

[17] Hirose I, Sano K, Shioda I, Kumano M, Nakamura K (2000). Proteome analysis of *Bacillus subtilis* extracellular proteins: a two-dimensional protein electrophoretic study.. Microbiology.

[18] Jeffery CJ (1999). Moonlighting proteins.. Trends Biochem Sci.

[19] Jeffery CJ (2003). Moonlighting proteins: old proteins learning new tricks.. Trends Genet.

[20] Huh WK, Falvo JV, Gerke LC, Carroll AS, Howson RW (2003). Global analysis of protein localization in budding yeast.. Nature.

[21] Gardy JL, Laird MR, Chen F, Rey S, Walsh CJ (2005). PSORTb v.2.0: expanded prediction of bacterial protein subcellular localization and insights gained from comparative proteome analysis.. Bioinformatics.

[22] Shen HB, Chou KC (2007). Gpos-PLoc: an ensemble classifier for predicting subcellular localization of Gram-positive bacterial proteins.. Protein Eng Des Sel.

[23] Bairoch A, Boeckmann B, Ferro S, Gasteiger E (2004). Swiss-Prot: juggling between evolution and stability.. Briefings in Bioinformatics.

[24] Zhou M, Boekhorst J, Francke C, Siezen RJ (2008). LocateP: genome-scale subcellular-location predictor for bacterial proteins.. BMC Bioinformatics.

[25] Emanuelsson O, Brunak S, von Heijne G, Nielsen H (2007). Locating proteins in the cell using TargetP, SignalP and related tools.. Nat Protoc.

[26] Bendtsen JD, Nielsen H, Von Heijne G, Brunak S (2004). Improved Prediction of Signal Peptides: SignalP 3.0.. J Mol Biol.

[27] Bendtsen JD, Nielsen H, Widdick D, Palmer T, Brunak S (2005). Prediction of twin-arginine signal peptides.. BMC Bioinformatics.

[28] Juncker AS, Willenbrock H, Von Heijne G, Brunak S, Nielsen H (2003). Prediction of lipoprotein signal peptides in Gram-negative bacteria.. Protein Sci.

[29] Bendtsen JD, Kiemer L, Fausboll A, Brunak S (2005). Non-classical protein secretion in bacteria.. BMC Microbiol.

[30] Gardy JL, Brinkman FS (2006). Methods for predicting bacterial protein subcellular localization.. Nat Rev Microbiol.

[31] Daffé M, Draper P (1998). The envelope layers of mycobacteria with reference to their pathogenicity.. Adv Microb Physiol.

[32] Barry CE (2001). Interpreting cell wall ‘virulence factors’ of *Mycobacterium tuberculosis*.. Trends Microbiol.

[33] Jarlier V, Nikaido H (1994). Mycobacterial cell wall: structure and role in natural resistance to antibiotics.. FEMS Microbiol Lett.

[34] Restrepo-Montoya D, Vizcaino C, Nino LF, Ocampo M, Patarroyo ME (2009). Validating subcellular localization prediction tools with mycobacterial proteins.. BMC Bioinformatics.

[35] Sonnhammer EL, von Heijne G, Krogh A (1998). A hidden Markov model for predicting transmembrane helices in protein sequences.. Proc Int Conf Intell Syst Mol Biol.

[36] Kall L, Krogh A, Sonnhammer EL (2004). A combined transmembrane topology and signal peptide prediction method.. J Mol Biol.

[37] Gu S, Chen J, Dobos KM, Bradbury EM, Belisle JT (2003). Comprehensive proteomic profiling of the membrane constituents of a *Mycobacterium tuberculosis* strain.. Mol Cell Proteomics.

[38] Sinha S, Kosalai K, Arora S, Namane A, Sharma P (2005). Immunogenic membrane-associated proteins of *Mycobacterium tuberculosis* revealed by proteomics.. Microbiology.

[39] Larsen JEP, Lund O, Nielsen M (2006). Improved method for predicting linear B-cell epitopes.. Immunome research.

[40] Rubinstein ND, Mayrose I, Pupko T (2009). A machine-learning approach for predicting B-cell epitopes.. Molecular Immunology.

[41] Malen H, Berven FS, Fladmark KE, Wiker HG (2007). Comprehensive analysis of exported proteins from *Mycobacterium tuberculosis* H37Rv.. Proteomics.

[42] Xiong Y, Chalmers MJ, Gao FP, Cross TA, Marshall AG (2005). Identification of *Mycobacterium tuberculosis* H37Rv integral membrane proteins by one-dimensional gel electrophoresis and liquid chromatography electrospray ionization tandem mass spectrometry.. J Proteome Res.

[43] De Smet KAL, Weston A, Brown IN, Young DB, Robertson BD (2000). Three pathways for trehalose biosynthesis in mycobacteria.. Microbiology.

[44] Fu LM, Fu-Liu CS (2007). The gene expression data of *Mycobacterium tuberculosis* based on Affymetrix gene chips provide insight into regulatory and hypothetical genes.. BMC microbiology.

[45] Jarling M, Cauvet T, Grundmeier M, Kuhnert K, Pape H (2004). Isolation of mak1 from Actinoplanes missouriensis and evidence that Pep2 from Streptomyces coelicolor is a maltokinase.. Journal of basic microbiology.

[46] Garg S, Alam MS, Bajpai R, Kishan KVR, Agrawal P (2009). Redox biology of *Mycobacterium tuberculosis* H37Rv: protein-protein interaction between GlgB and WhiB1 involves exchange of thiol-disulfide.. BMC Biochemistry.

[47] Garg SK, Alam MS, Kishan KVR, Agrawal P (2007). Expression and characterization of alpha-(1, 4)-glucan branching enzyme Rv1326c of *Mycobacterium tuberculosis* H37Rv.. Protein expression and purification.

[48] Deleage G, Combet C, Blanchet C, Geourjon C (2001). ANTHEPROT: an integrated protein sequence analysis software with client/server capabilities.. Computers in biology and medicine.

[49] Sutcliffe IC, Harrington DJ (2004). Lipoproteins of *Mycobacterium tuberculosis*: an abundant and functionally diverse class of cell envelope components.. FEMS Microbiol Rev.

[50] Narita S, Tokuda H (2007). Amino acids at positions 3 and 4 determine the membrane specificity of *Pseudomonas aeruginosa* lipoproteins.. J Biol Chem.

[51] Robichon C, Bonhivers M, Pugsley AP (2003). An intramolecular disulphide bond reduces the efficacy of a lipoprotein plasma membrane sorting signal.. Mol Microbiol.

[52] Andersen P, Askgaard D, Ljungqvist L, Bennedsen J, Heron I (1991). Proteins released from *Mycobacterium tuberculosis* during growth.. Infect Immun.

[53] Mawuenyega KG, Forst CV, Dobos KM, Belisle JT, Chen J (2005). *Mycobacterium tuberculosis* functional network analysis by global subcellular protein profiling.. Mol Biol Cell.

[54] Converse SE, Cox JS (2005). A protein secretion pathway critical for *Mycobacterium tuberculosis* virulence is conserved and functional in Mycobacterium smegmatis.. J Bacteriol.

[55] Casali N, White AM, Riley LW (2006). Regulation of the *Mycobacterium tuberculosis* mce1 operon.. J Bacteriol.

[56] Shimono N, Morici L, Casali N, Cantrell S, Sidders B (2003). Hypervirulent mutant of *Mycobacterium tuberculosis* resulting from disruption of the mce1 operon.. Proc Natl Acad Sci U S A.

[57] Liang S, Zheng D, Zhang C, Zacharias M (2009). Prediction of antigenic epitopes on protein surfaces by consensus scoring.. BMC Bioinformatics.

[58] Sinha S, Arora S, Kosalai K, Namane A, Pym AS (2002). Proteome analysis of the plasma membrane of *Mycobacterium tuberculosis*.. Comp Funct Genomics.

[59] Houghten RA (1985). General method for the rapid solid-phase synthesis of large numbers of peptides: specificity of antigen-antibody interaction at the level of individual amino acids.. Proc Natl Acad Sci U S A.

[60] Song H, Sandie R, Wang Y, Andrade-Navarro MA, Niederweis M (2008). Identification of outer membrane proteins of *Mycobacterium tuberculosis*.. Tuberculosis (Edinb).

[61] Smith PK, Krohn RI, Hermanson GT, Mallia AK, Gartner FH (1985). Measurement of protein using bicinchoninic acid.. Anal Biochem.

[62] Hirschfield GR, McNeil M, Brennan PJ (1990). Peptidoglycan-associated polypeptides of *Mycobacterium tuberculosis*.. J Bacteriol.

[63] Rezwan M, Laneelle MA, Sander P, Daffe M (2007). Breaking down the wall: fractionation of mycobacteria.. J Microbiol Methods.

[64] Wagner B, Fattorini L, Wagner M, Jin SH, Stracke R (1995). Antigenic properties and immunoelectron microscopic localization of *Mycobacterium fortuitum* beta-lactamase.. Antimicrob Agents Chemother.

